# Epidemiology Without Biology: False Paradigms, Unfounded Assumptions, and Specious Statistics in Radiation Science (with Commentaries by Inge Schmitz-Feuerhake and Christopher Busby and a Reply by the Authors)

**DOI:** 10.1007/s13752-016-0244-4

**Published:** 2016-06-17

**Authors:** Bill Sacks, Gregory Meyerson, Jeffry A. Siegel

**Affiliations:** Center for Devices and Radiological Health, U.S. Food and Drug Administration, Green Valley, AZ USA; Department of English, North Carolina Agricultural and Technical State University, Greensboro, NC USA; Nuclear Physics Enterprises, Marlton, NJ USA

**Keywords:** Adaptive response, Biology, Hormesis, Linear no-threshold, Paradigm, Radiation, Radiophobia

## Abstract

Radiation science is dominated by a paradigm based on an assumption without empirical foundation. Known as the linear no-threshold (LNT) hypothesis, it holds that all ionizing radiation is harmful no matter how low the dose or dose rate. Epidemiological studies that claim to confirm LNT either neglect experimental and/or observational discoveries at the cellular, tissue, and organismal levels, or mention them only to distort or dismiss them. The appearance of validity in these studies rests on circular reasoning, cherry picking, faulty experimental design, and/or misleading inferences from weak statistical evidence. In contrast, studies based on biological discoveries demonstrate the reality of hormesis: the stimulation of biological responses that defend the organism against damage from environmental agents. Normal metabolic processes are far more damaging than all but the most extreme exposures to radiation. However, evolution has provided all extant plants and animals with defenses that repair such damage or remove the damaged cells, conferring on the organism even greater ability to defend against subsequent damage. Editors of medical journals now admit that perhaps half of the scientific literature may be untrue. Radiation science falls into that category. Belief in LNT informs the practice of radiology, radiation regulatory policies, and popular culture through the media. The result is mass radiophobia and harmful outcomes, including forced relocations of populations near nuclear power plant accidents, reluctance to avail oneself of needed medical imaging studies, and aversion to nuclear energy—all unwarranted and all harmful to millions of people.

## Introduction^1^

Paradigms, although absolutely necessary for our interpretation of nature, can either help or hinder our understanding of reality. As Kuhn discussed in his groundbreaking 1962 book *The Structure of Scientific Revolutions* (1996), when a new paradigm arises as a result of a scientific revolution and then becomes established as “normal science,” it guides the accumulation of discoveries in its particular branch of science. But as scientific investigation proceeds under a particular paradigm, with proliferating discoveries that appear to confirm it and that are regarded as “signal,” other discoveries will often accumulate that appear to counter the now-prevailing paradigm. These contrary discoveries are at first regarded as “noise,” to be put aside and dealt with later. But often they are not “dealt with later,” and instead are ignored for protracted periods of time. This delay is often produced by influences outside the fields of science and instead comes from politically powerful institutions, whether the church, governmental agencies, or professional organizations.

Sooner or later, however, the proliferation of noise begins to outpace the accumulation of signal, and an alternative paradigm—either new or perhaps one that was expressed earlier but stayed eclipsed for some time—takes the stage and begins to contend for supremacy. During this process, which can be fairly prolonged, the competing paradigm purports to explain all those discoveries previously explained by the prevailing one plus the accumulated counter-discoveries, turning the latter from noise into signal, and much of the erstwhile signal into noise or into outright erroneous perception.

This phenomenon is epitomized in radiation protection science by the prevailing linear no-threshold (LNT) paradigm of radiation carcinogenesis—initially a gigantic scientific oversight that was taken over as a policy choice and now masquerades as a scientific discovery (explained more fully in the section after next). The LNT paradigm, extrapolating putative low-dose effects down from effects at high doses of ionizing radiation, where it has a legitimate claim to validity, assumes and asserts, without evidence, two things: first, that *all* acute exposures to ionizing radiation are harmful and proportional to that dose, *regardless of how low* the dose, all the way down to zero exposure; and second, that this effect is *cumulative* over one’s lifetime, regardless of how low the *rate of delivery of that dose (dose rate)*.

A mathematical corollary of the proportionality (linearity) concept, known as “collective dose,” is that the same dose shared by any number of people will cause the same number of cancers and/or deaths from cancer—i.e., the same total dose will produce, say, 10 cancers whether it is received by 10,000 or 100,000 people. This is analogous to observing that if a person takes 100 aspirins at one time there will be a single death, and then asserting that the same single death will occur on average as a result of 100 persons each taking one aspirin—in other words, it is like claiming that no matter how the 100 person-aspirins is distributed, the resulting number of deaths will be the same on average. Since we know that a single aspirin will not, in general, produce a single death even in 100 people, there must be something wrong with the expectation.

Our review examines this LNT paradigm that presently governs almost all radiation-related regulatory policy in the world and that informs a significant number of putatively scientific, peer-reviewed papers, but is now also facing significant opposition. As we show, LNT is characterized by a one-sided failure to consider adequately the basic experimental sciences of biology, physics, chemistry, and others, in favor of a sterile epidemiology, rooted in a misuse of mathematics and statistics designed to confirm a priori conclusions. The one-sidedness lies in focusing only on the unquestioned molecular damage, while ignoring the biological response of the organism.

To be clear, the problem with the LNT paradigm is not that its predictions are totally illegitimate in all dose ranges, because they are not. Rather the problem is that, by categorically denying the existence of a threshold below which harm is absent, LNT is being tenaciously applied outside its domain of applicability—in the low-dose and low-dose-rate range. To put it another way, the LNT paradigm is not wholly fanciful *even in the low*-*dose and low*-*dose*-*rate domain*. Rather it is *incomplete*. In effect, it regards the DNA, the cell, the tissue, and the organism as passive recipients of their own radiation-produced molecular damage. It further regards each of these levels of organization as an isolated system with no relationship to its surroundings other than to the incoming radiation (ionizing will be understood hereafter), in particular with no relationship to the rest of the cell, its neighbors, or the organism as a whole. In short, it is a particular form of reductionism.

The large school of scientific papers, albeit not the majority, that fail in this regard have been shoehorned into place as the conventional wisdom by regulatory policies and agencies throughout most of the world, supported by its percolation upward into popular culture through one-sided media attention. This school sidetracks the more numerous studies that are based in experimental biology but that are rarely if ever consulted or cited by the main-track school. It is difficult to gain a sense of the relative number of papers within each school, though attempts at compiling lists of the biologically based papers have been made by Luckey in his book *Radiation Hormesis* (1991) and Sanders in his book *Radiation Hormesis and the Linear*-*No*-*Threshold Assumption* (2010). The literature on radiation carcinogenesis and on radiation hormesis is vast and continues to grow (ACMUI 2015), and even a cursory review of this literature is beyond the scope of this review. However, in order to provide some context for this ongoing controversy, many notable studies are reviewed in the present article.

The LNT paradigm often leads its proponents to commit egregious errors of logic and inference. Detailed attempts to expose these errors sometimes encounter difficulty getting published in journals, which only serves to buttress the fallacious modes of thinking by granting them safe harbor. The resulting impact at the level of policy and peer-reviewed science is at least as damaging when it then lends unwarranted credence to distortions at the level of popular culture.

We will discuss, in closing, three major classes of events exemplifying the consequences of such distortions: First, the unnecessary and deadly forced relocations of immense numbers of people near nuclear power plant accidents. Second, a growing fear-driven refusal by many patients and parents to allow themselves or their children to undergo potentially life-saving radiological imaging studies—CT scans and plain X-rays. And third, energy proposals that characterize the radiophobic anti-nuclear environmental movement and that spread fear-uncertainty-doubt (FUD) in popular media.

## Background: The Untrustworthiness of Most Medical Journal Papers

It is no news to anyone paying attention that editors of certain medical journals have begun to admit that many of the papers contained in their publications contain largely irreproducible results or conclusions that are just plain wrong. Dr. Richard Horton, editor in chief of *The Lancet*, recently stated:

Much of the scientific literature, perhaps half, may simply be untrue. Afflicted by studies with…*an obsession for pursuing fashionable trends of dubious importance*, science has taken a turn towards darkness…In their quest for telling a compelling story, scientists too often *sculpt data to fit their preferred theory of the world*…Journal editors deserve their fair share of criticism too. We aid and abet the worst behaviours….Our love of “significance” pollutes the literature with many a statistical fairy-tale. We reject important confirmations….And individual scientists, including their most senior leaders, do little to alter a research culture that occasionally veers close to misconduct. (Horton 2015, p. 1380; emphasis added)

Dr. Marcia Angell wrote some years ago:

It is simply no longer possible to believe much of the clinical research that is published, or to rely on the judgment of trusted physicians or authoritative medical guidelines. I take no pleasure in this conclusion, which I reached slowly and reluctantly over my two decades as an editor of *The* *New England Journal of Medicine*. (Angell 2009)

The much-quoted Dr. John Ioannidis, Stanford professor of medicine and health research and policy, has been exposing and criticizing the prevalence of such fallacious literature for many years (Ioannidis 2010).

But to know that even a majority of scientific papers are in error for one reason or another is not to know which ones are fallacious or what to do about this state of affairs. Such knowledge is valuable only insofar as it leads to a search for ways to tell which scientific papers are valid and which are not.

One way to distinguish between these categories is to look for that set of studies that converge on a single consistent theoretical outlook by adducing many lines of evidence. The alternative consists of studies that fail to provide converging lines of evidence and that often repeatedly commit the same methodological errors. Furthermore, the latter fail to refute the various lines of evidence discovered and revealed by the former set, and instead either distort or neglect altogether this evidence (Shermer 2015). Here we point to such erroneous efforts in radiation protection science (hereafter referred to as simply radiation science).

First we should examine the validity of the paradigm under which authors are operating. Examination of specific errors by specific authors, in addition to the paradigmatic ones, may also produce useful general lessons for other studies, since they tend to be repeated. It is unlikely that the particular authors whom we criticize in this review would disagree with Horton’s, Angell’s, or Ioannidis’ *general* assessment, but they seem unaware of their own contribution to this harmful state of affairs. Indeed we all have to remain continually aware of our own susceptibility to unsupported and unquestioned assumptions.

In this review we extend the ongoing critique of the current state of medical science by examining the state of radiation science in some depth. In particular, we attempt to expose the disjunction between the basic sciences of biology, physics, and chemistry, on the one hand, and a class of papers that confine their investigations to epidemiological, mathematical, and statistical considerations without reference to the basic sciences, or that refer to them only to dismiss or distort their well-established findings. This separation of the epidemiological from the biological, and/or, within biology, the separation of damage from the biological response to that damage, are central components of a major paradigmatic error. It yields a class of studies that invoke LNT as an a priori assumption and, based on circular reasoning, arrive at a self-fulfilling conclusion that LNT is valid, and then present the “measured” slope of the assumed dose–response relationship as a fact that is then uncritically and repeatedly cited.

## The LNT Paradigm of Radiation Carcinogenesis Explained in Greater Depth

Although radiation is known to cause cancer at high doses and high dose rates (i.e., high doses of radiation delivered over short time durations, rather than over protracted intervals such as is experienced with continual chronic radiation from natural background sources), there are no data to support this connection at low doses and dose rates (<100–200 mSv acute or chronic exposures; a mSv, or millisievert, is a unit of radiation dose, closely related to a mGy, or milligray^2^). In the absence of data, a hypothetical model must be therefore derived from high-dose data to estimate what the presumed carcinogenic effects of low-dose radiation might be. The most commonly employed model is the LNT model wherein dose–effect data at high doses are simply extrapolated linearly downward to zero dose with no threshold. The LNT model—although heavily promoted by scientific advisory bodies around the world and serving as the established paradigm used by radiation regulators—is demonstrably wrong, and its use for estimation of cancer risks resulting from low-dose radiation exposures is unjustifiable (Siegel and Stabin 2012; Siegel and Pennington 2015).

We are literally bathed every second of every day in low-dose-rate radiation from natural background: there is an average exposure of 3 mSv per year in the U.S., ranging up to 260 mSv per year on the rest of the planet depending upon where one lives. For comparison, a computed tomography (CT) medical imaging scan is associated with an acute radiation dose of approximately 10 mGy, well within the observed range of annual natural background exposures. Irrespective of the level of natural background or other low-dose and/or low-dose-rate exposure to a given population, no associated health effects have been documented anywhere in the world.

The overriding fallacy embodied in the LNT model is that it ignores the fact that the body responds differently to radiation at high versus low acute doses and dose rates, as has been demonstrated in many studies: high-dose exposures are associated with inhibition of protective responses and extensive damage to the organism, while at low doses the body eliminates the damage through a variety of protective mechanisms, evolved in humans from eons of living in a world bathed in natural background radiation.

When considering a broad, organismal-level perspective, the pitfalls of the LNT model of radiation carcinogenesis become apparent. For example, *the spontaneous rate ofDNAalterations due to the normal oxidative metabolic processes in our cells dwarfs theDNAalteration rate due to background or most other radiation exposures* (Billen 1990; Siegel and Welsh 2015). The natural background radiation mutation rate, assuming an average background exposure rate of 3 mSv per year in the U.S. (lower than in many regions of the world), would be 3–30 DNA alterations per cell per year, which is almost 2.5 million times lower than the spontaneous mutation rate due to normal metabolism. Background exposure rates even a hundred times greater would still produce DNA alterations several orders of magnitude lower than those due to normal metabolism. The point is that the normal body effectively deals with these numerous spontaneous mutations through a set of mechanisms collectively called the adaptive response; the small excess conferred by a low dose of radiation, even if LNT were true, would not likely be detectable. We will provide compelling evidence that the dose–effect relationship at low doses is not linear, and that there is an obvious threshold reflecting and demonstrating the existence of the body’s adaptive protective responses.

## The Adaptive Response: Known Biological Mechanisms of Repair and Defense Against Low Levels of Radiation

Although any *damage* that may occur after exposure to low-dose radiation may happen in a linear fashion (i.e., the dose-damage response may be linear), the net dose–response at this dose level is not linear because of the body’s demonstrated response to mitigate or eliminate this damage. There is much experimental evidence supporting the induction of adaptive protection against cancer, such as antioxidant production, apoptosis, immune system-mediated effects, and repair of DNA double-strand breaks that have been shown to occur even after patient exposure to the low-dose radiation from CT scans (Löbrich et al. 2005).

DNA damage response mechanisms defend against exogenous and endogenous DNA damage and enhance both survival and maintenance of genomic stability (which is critical for cancer avoidance). Importantly, as noted above, the spontaneous rate of DNA alterations absolutely dwarfs the DNA alteration rate due to background radiation (Billen 1990; Siegel and Welsh 2015). It must be noted that the vast majority of human cancers are not simply the end product of one or more mutations. Such mutations may be necessary, but they are not sufficient to produce cancer. The 2015 Nobel Prize in Chemistry went to three investigators—Lindahl, Modrich, and Sancar—for discovering three intracellular repair mechanisms that prevent most of us from getting cancer on a regular basis. In addition to intracellular DNA repair mechanisms, modern understanding of the role of the immune system in the development of clinically overt cancers has led to a replacement of the outdated “one mutation = one cancer” model. In fact, deficiencies in repair enzymes and/or evasion from immune system detection and destruction have emerged as the newest explanations for cancer formation, rather than simply DNA damage.

Numerous laboratory investigations of cellular and organismal responses to low-dose and low-dose-rate radiation have led to the discovery of at least six different mechanisms that account for lower rates of cancer and greater longevity in humans and in many other animals. These beneficial outcomes result from the stimulation, by low levels of damage to an organism’s constituent parts, of a set of biological responses collectively referred to as *hormesis* (from the Greek for stimulating, as in hormone) (Miller et al. 1989; Luckey 1991; Sponsler and Cameron 2005; Sanders 2010; Neumaier et al. 2011; Cuttler and Sanders 2015).

These response mechanisms include, with possibly more yet to be discovered,

1. enhanced production in the cell’s nucleus of repair enzymes for damaged DNA,
2. slowed mitosis that permits these enzymes to accomplish their function,
3. induced apoptosis that destroys cells that escape the repair,
4. enhanced production of antioxidant enzymes that lower the rate of damage to DNA and other molecules even from normal metabolic mitochondrial production of reactive oxygen species (ROS)—continual damage outweighing that from radiation by several orders of magnitude,
5. bystander effects, in which neighboring unexposed or undamaged cells trade chemical messengers that enhance apoptosis in cells with unrepaired or misrepaired DNA, and
6. enhanced immune surveillance and removal of cells that fail to repair themselves or to undergo apoptosis.

Thus reductionism fails to consider the organism as an entire system in which there are several layers of defense against radiation damage that evolution, at least through the agency of natural selection, has provided to animal (e.g., human) cells and organisms, all working in concert from the more local (cellular) layers up to the systemic (immune system).

Furthermore, hormesis is a very general phenomenon among living organisms. It entails the existence of at least two and often three domains within the dose scale—too little, best, and too much for optimal health—in response to impacting agents. Examples include everyday physical, chemical, or other types of agents such as sunlight, water, oxygen, wine, vitamins, fear, and countless others. Each of these comes in doses that are either too little, just right, or too much. Radiation is no exception, and the burden of proof should rightly fall on the claim that it is the exception, not on the claim that it is like so many other agents. Yet the paradigm turns this around. As Carl Sagan once said, “Extraordinary claims require extraordinary evidence.”

## Evolution: The Most Important Feature of Biology

Adherence to LNT ignores the well-established fact that ever since life began—some 3 billion years ago on this 4.5 billion-year-old Earth—it has been evolving through the primary, though not sole, mechanism of natural selection. Often ignored too is the fact that radioactivity from heavy elements, created in supernovae and present since the formation of the solar system, constitutes a veritable sea of radiation in our earthly environment. Indeed radioactivity accounts for the dominant portion of the heat generation within the Earth that maintains much of the core in a liquid state and the mantle sufficiently plastic to keep tectonic plates moving on the Earth’s surface.

Since radioactive elements decay over time, the radioactivity has also been declining throughout the Earth’s life, particularly during the 3 billion years of the biosphere. This means that when life began, the amount of radioactivity from the ground was a good deal more intense than it is now. Furthermore, this intensity varies from place to place on the planetary surface by more than two orders of magnitude, exposing local inhabitants to significantly varying amounts of natural background radiation.

Bathed in this sea of radiation throughout their evolution, species of life forms have been forced to adapt or become extinct. Only those that have adapted through the development of protective responses against damaging radiation have survived this natural selection, whether they are bacteria, fungi, plants, or animals. These responses have been bequeathed to all extant species, including humans. Any field of science that ignores, dismisses, or distorts this reality—particularly one that calls itself radio*biology*, or radiation *biology*—relinquishes its claim to validity.

In contrast to the LNT paradigm’s insistence that all radiation is harmful and the harm is cumulative, no matter how low the dose or dose rate, the school of radiation science that is based in evolutionary biology and recognizes the very widespread phenomenon of hormesis holds that low-dose and low-dose-rate radiation stimulates a set of biological responses in organisms that not only repair and defend against the radiogenic damage, *but do so in excess of immediate need*, so that they enhance protections even against other current and future sources of damage—including subsequent higher radiation exposures, infections, and, most importantly, against the ubiquitous intracellular reactive oxygen species (ROS) that are the byproducts of normal metabolism (Feinendegen et al. 2012).

Furthermore, linearity in biology is generally, if not always, a figment of the mathematical imagination in search of ease in calculation or aesthetic appeal. It rarely if ever exists in living matter, or in science in general, *beyond limited domains in which linearity is approximated* and beyond which *non*linearity becomes the dominant feature. Nonlinearity occurs because of complex interactions among multifarious biological or other processes that come into play under various conditions and at various levels of organization.

## A Brief History of the Introduction of the LNT Fiction into Science

We have described elsewhere (Siegel et al. 2015a) how Hermann Muller—winner of the 1946 Nobel Prize in Physiology or Medicine for his work on radiation-caused mutations in fruit flies—and his colleagues failed to see that their data demonstrated a threshold with respect at least to dose *rate*, even though their experimental doses were mainly in the high-dose range. Having earlier failed to realize the import of their own data, Muller announced to the world during his Stockholm acceptance speech that there was no escape from the conclusion that radiation harms linearly down to zero dose, regardless of dose rate. Thus LNT was forcefully injected by a prestigious scientist into the field of radiation research and regulation, with no significant objection by any scientists at the time. It has been firmly ensconced ever since, and has become even more so over time.

What was really linearity at high doses with a threshold at lower doses, below which there is no harm or excess mutations in fruit flies—what might be termed LT for linear-threshold, or linear-down-to-a-threshold—became LNT for linear *no*-threshold, all because of an immense scientific blind spot due to the developing and strengthening paradigm of harm at any dose or dose rate. While this may or may not have represented unwitting paradigm blindness on the part of many scientists, regulatory agencies and advisory groups, in contrast, picked up on this paradigm, and *knowingly* turned it to their own advantage (Calabrese 2015). In the 1950s LNT was equally falsely applied to the specific harm of cancer causation, and has remained there ever since (Lewis 1957).

Many now attempt to justify their assumptions by reasons they consider practical. Leading figures in the radiation protection field now go beyond their admission that LNT is mere assumption to justify it as either the most “plausible” fit to the data (Boice 2015) or that the linear model differs insignificantly from the better fit by a curved line (e.g., linear quadratic), and therefore, since the straight line is more convenient mathematically, there is no reason not to retain it (Leuraud et al. 2015).

Further, a recent update to the Life Span Study (LSS) atomic-bomb survivor data, considered to be the gold standard of dose–response data, indicated that the revised data for cancer mortality at low doses are more consistent with a linear-quadratic dose–response model because a significant upward curvature is exhibited (Ozasa et al. 2012). Use of a more generalized model employing multiple linear regression indicated the presence of a nonzero dose threshold, and in addition, when a correction was applied to these data for a likely bias in the baseline cancer rate, it provided possible evidence of radiation hormesis (Doss 2013). That is, excess relative risk (ERR) values were negative for all doses below approximately 0.6 Gy (or 600 mGy). This is indicative of a beneficial or cancer-preventative effect such that low-dose radiation would reduce rather than increase cancer risk when compared with the risk in an environment with even lower dose or dose-rate; that is, the slope of the response-versus-dose graph is negative in the very low-dose range. Another recent reanalysis of the LSS cohort of A-bomb survivors using a nonparametric statistical procedure has revealed a threshold around 0.2 Sv (or 200 mSv), below which the response is manifested as a negative ERR, again consistent with a radiation hormesis model (Sasaki et al. 2014). The epidemiologically observed threshold and negative ERRs are in agreement with experimental evidence of adaptive protection against cancer at low doses, as exemplified by enhanced repair of DNA double-strand breaks, increased antioxidant production, stimulated apoptosis, and upregulated immune system surveillance and removal of malignant cells.

A second justification generally given for the LNT assumption is the belief that it errs on the safe side, a devastating one-sided illusion that we discuss below.

## Errors of Biological Commission and Omission in Radiation Science

The paradigm that misrepresents or neglects the science surrounding biological responses has become so prevalent within the (thus misnamed) field of radio*biology* that it blinds its advocates, and its unwitting acolytes, not only to reality but even to the generally accepted rules of scientific inference, as we show below in our discussion of a paper by Leuraud et al. (2015).

This biological neglect comes in two forms—errors of commission and errors of omission.

### Errors of Biological Commission

There are two types of errors of biological commission: mention only to dismiss and mention that distorts the science.

#### Mention only to Dismiss

##### Hall and Brenner

An example is a 2004 response by Hall and Brenner (2004), leading advocates in the field of radiology of the proposition that all radiation is harmful, to a letter by Welsh (2004), a radiation oncologist, in which Welsh ventured an evolutionary biologist’s explanation for the phenomenon of hormesis. He explained that over the billions of years on Earth biological entities have adapted by evolving protective responses to damage not only from radiation, but from a wide variety of agents to which they have been exposed in the natural environment of the biosphere—whether these entities be physical, chemical, or any other potentially damaging aspect of our shared environment.

In response Hall and Brenner stated, “Dr. Welsh really misses the point when he proposes *biologic* explanations of why most very-low-dose radiation *epidemiologic* studies show little or no effect” (2004; emphasis added). Welsh’s point was that there is every reason to consider evolutionary *biological*—cellular and immunological—processes to explain well-known laboratory-proven *defenses and protections* against low-dose radiation. He offered this not as an explanation of nondetectability of effect, but rather as a much-neglected description of scientific reality. It was Hall and Brenner who missed Welsh’s point. In fact, they explicitly *argue against* appeals to biology in favor of considerations of poor signal-to-noise ratios (a statistical issue) as the explanation for the nondetectability of harm. They write, “When the ratio of signal (radiation risk) to noise (background risk) [more correctly, the *variability* in background risk] is small, one expects inconclusive results from necessarily low-powered epidemiologic studies, *purely because of the statistics*” (Hall and Brenner 2004; emphasis added).

By explaining the nondetectability of harm not by its absence but rather by its noisy camouflage, they shield themselves from having to admit that there may be an actual *absence* of harm at low doses, though at the same time *they remove any doubt that their assertion of harm at low doses is merely assumed and not based in evidence*.

Interestingly, Hall and Brenner’s dismissal itself exemplifies a signal-to-noise problem, in which they reverse signal and noise by regarding Welsh’s contention as biological noise against the background of what they regard as epidemiological signal—from the continual flux of studies that are disembodied from biological reality. When a paradigm, along with the resultant espousals and reputations, so firmly grips the mind, then anything that derives from an alternative outlook is perceived as noise that obscures the presumed signal.

##### Boice

John Boice, the current president of the National Council on Radiation Protection and Measurements (NCRP) and a health physicist and radiation epidemiologist, in his monthly column in the *Health Physics News* of September 2015 titled “LNT 101” states that

Epidemiology is an observational (i.e., non-experimental) science. It is not possible to provide convincing and consistent evidence of risks in the low-dose domain because of the inability to control for confounding factors and biases as well as *the statistical inability to detect a tiny signal against a huge background noise* (i.e., cancer is not an uncommon disease); the inherent uncertainties are just too great. (Boice 2015, p. 26; emphasis added)

##### The BEIR VII Report

The National Academy of Sciences’ committee known as BEIR (Biological Effects of Ionizing Radiation) has issued a very influential series of reports over the years that authors who subscribe to LNT often refer to as the standard of truth. The latest is the BEIR VII report (2006). Repeating the nondetectability mantra, BEIR VII does not believe an excess cancer rate of 1–2 % is detectable and states in Appendix D: Hormesis:

Another important consideration is the expected magnitude of the increase in health effect induced by excess background radiation. If one assumes a LNT response, a calculation can be made for expected cancers induced by excess radiation in a high-background-radiation area. As an example, consider the elevated levels of gamma radiation in Guodong Province, Peoples’ Republic of China (PRC). In this study, a population receiving 3–4 mGy per year was compared to an adjacent control population receiving 1 mGy per year. No difference in cancers was noted between the high-background area and the control area (NRC 1990). One can estimate the expected excess percentage of cancers resulting from the 2–3 mGy difference in exposure per year using a linear nonthreshold model and the lifetime risk estimates developed in this report. A calculation by this committee indicated that the expected percentage of cancers induced by the excess background radiation would be 1–2 % above the cancers occurring from all other causes in a lifetime. Even if all confounding factors were accounted for, it is questionable whether one could detect an excess cancer rate of 1–2 %. *Excess cancers may indeed be induced by elevated radiation exposure in high*-*background areas, but the excess may not be detectable given the high lifetime occurrence of cancer from all causes*. (BEIR VII 2006, p. 335; emphasis added)

#### Mention that Distorts the Science

##### The BEIR VII Report

Undeterred by even their own admitted nondetectability in the low-dose range, BEIR VII nevertheless delves into biological considerations. But the committee does so only in order to demonstrate the reality of LNT, i.e., the absence of a threshold (the NT part of LNT) below which there is no harm from radiation. They do this in order to explain away the voluminous laboratory findings to the contrary and show why they cannot be true, thereby reinforcing belief in LNT. In so doing they merely dismiss much of the work in such research, particularly research showing that normal metabolic processes, through the creation of reactive oxygen species in mitochondria, do several orders of magnitude more damage to DNA than does radiation.

In particular, in Chapter 1 BEIR VII grants the existence of repair but contends, without evidence, that repair *is incomplete* (BEIR VII 2006, Chap. 1). Before discussing that contention further, we note that the assertion that even incomplete repair would exhibit *linearity* down to zero is an ad hoc rationalization that entails the denial that there are qualitatively different bodily responses at different dose ranges. However, as noted above, much evidence indicates that there most certainly are such responses. Thus this salvaging attempt, by the invocation of incompleteness of repair, fails as an explanation of (assumed) linearity.

The BEIR VII (2006) report simply asserts on page 246:

Mechanistic uncertainties remain, but the weight of available evidence would argue against the presence of a low dose threshold for tumor induction based on error-free repair of initial DNA damage. In summary, the committee judges that the balance of scientific evidence at low doses tends to weigh in favor of a simple proportionate relationship between radiation dose and cancer risk.

Furthermore, as we show below, the BEIR committee engaged in severe cherry picking to support this contention. To be clear, cherry picking is not merely being selective. All authors of writing in any subject whatsoever are necessarily selective. Otherwise no paper, essay, or book would be of finite length. But cherry picking is a special form of selectivity. Its essence lies not in what is chosen for inclusion, but rather what is chosen for *exclusion*—whether deliberately or unwittingly—and the consequent ways in which the inclusions are treated. Cherry picking involves selective exclusion of irrefutable evidence that contradicts the cherry pickers’ contentions.

The report explicitly recognizes that a curved line fits better than a straight line for certain dose–response radiation data. Nevertheless the authors approximate that curve discontinuously by not one but two straight lines—one in the higher-dose region and a different one with a lower slope tangent to the lower-dose region—based on the use of a device called the dose and dose rate effectiveness factor (DDREF). This provides a means of modifying the linear model in order to preserve linearity. That this artifice ignores reality is evidenced by the LSS atomic-bomb survivor population, which does not exhibit a linear relationship at doses <100 mGy (Siegel et al. 2015c; Siegel and Welsh 2015). This renders the claim of low-dose linearity false and the appeal to DDREF scientifically meaningless. Linearity at low doses does not exist; rather, it is forced by the high-dose extrapolation of the LNT model. Thus the BEIR VII committee refuses to loosen their grip on linearity, maintaining it with a slight modification that misleadingly suggests an elevated level of sophistication. In addition to determined adherence to linearity, the committee forces the slope of their straight line in the low-dose region to be positive—by nothing other than assumption.

Furthermore, while the BEIR VII report mentions repair, it omits mention of other possible mechanisms of defense against damage from radiation that take place when repair fails. As listed above, these include apoptosis (cell suicide), bystander effects by messenger molecules exchanged between the damaged cell and its neighbors, and immune system cleanup of damaged unrepaired cells—all of which fail to save the damaged cells but protect the organism.

The BEIR VII report in Appendix D: Hormesis notes on page 332 that the evidence for a repair mechanism that acts to reduce both spontaneous and radiation-induced damage to below spontaneous levels, thus causing a hormetic effect, is weak and indirect and is contradicted by direct measures of DSB (double-strand breaks) repair foci at low doses. For this conclusion they cite a study by Rothkamm and Löbrich (2003).

However, the BEIR VII report misrepresents the cited reference, as this study actually comes to the opposite conclusion when not cherry picked. The report (BEIR VII 2006, p. 332) quotes from the abstract of the cited paper:

Surprisingly, DSBs induced in cultures of nondividing primary human fibroblasts by very low radiation doses (approximately 1 mGy) remain unrepaired…

but omits the words that immediately follow in the same sentence (Rothkamm and Löbrich 2003, p. 5057):

…for many days…

and that same sentence goes on to say:

…in strong contrast to efficient DSB repair that is observed at higher doses.

Thus Rothkamm and Löbrich suggest that at doses that are too low, repair is less efficient than at somewhat higher doses, doses that are still within the hormetic range but closer to the optimal level for such repair.

Furthermore, the next sentence in the abstract of the cited paper reads:

However, the level of DSBs in irradiated cultures decreases to that of unirradiated cell cultures *if the cells are allowed to proliferate after irradiation*, and we present evidence that this effect may be caused by an elimination of the cells carrying unrepaired DSBs…. (Rothkamm and Löbrich 2003, p. 5057; emphasis added)

Thus the paper mentions still other methods of defending the organism against radiation-caused damage, namely elimination of unrepaired cells, which include the three protective mechanisms described above.

The quoted abstract continues:

The results presented are in contrast to current models of risk assessment that assume that cellular responses are equally efficient at low and high doses…. (Rothkamm and Löbrich 2003, p. 5057)

Thus, Rothkamm and Löbrich point out that there are qualitatively different mechanisms that take place at low and high dose ranges with different efficiencies. So, far from supporting BEIR VIIs conclusion (2006, p. 323)—that “the current scientific evidence is consistent with the hypothesis that there is a linear, no-threshold dose–response relationship between exposure to ionizing radiation and the development of cancer in humans”—one of their own chosen citations stands in stark opposition to this conclusion when those portions that the report leaves unquoted are brought into view.

##### NRCs ACMUI Committee

The Nuclear Regulatory Commission’s (NRC) Advisory Committee on the Medical Uses of Isotopes (ACMUI) recently issued a report concerning three recent petitions for rulemaking submitted to the NRC (ACMUI 2015; NRC 2015). These petitions requested that the NRC amend its regulations and change the basis of those regulations from the LNT model of radiation protection to the radiation hormesis model. The ACMUI report recommended that

… in *the absence of definitive refutation of the LNT model* and while strongly encouraging continued investigation critically comparing alternative models, regulatory authorities should exercise prudent (though not excessive) conservatism in formulating radiation protection standards. The ACMUI therefore recommends that, for the time being and subject to reconsideration as additional scientific evidence becomes available, the NRC continue to base the formulation of radiation protection standards on the LNT model. (2015, p. 1; emphasis added)

In this statement ACMUI asserted that the burden of proof belongs to the “definitive refutation of the LNT model.” While such “definitive refutation” is present in countless studies, this raises the incidental question of *who really bears the burden of proof* and how such a question should be decided. Since the predominance of biological evidence is in favor of a threshold and much of it is in favor of hormesis below that threshold, why should the burden of proof not be on those who favor LNT? This is just another form of proof by assumption-and-assertion that substitutes for appeals to biology. And in this case, coming from an official committee, it serves to intimidate opponents.

Moreover, even if it is not explicitly stated, as it is in this quote from ACMUI, the mere assumption that LNT is true in effect anoints LNT as the null hypothesis and shifts the burden of proof to those who would deny any effect or a salutary one below a threshold. This is a misuse of the concept of a null hypothesis, which is a straw man designed to be rejected, if the data permit. A null hypothesis is not properly designed to stand as a challenge to one’s opponent that must be *accepted* as true if the opponent’s study lacks sufficient statistical power to reject it. A null is never *accepted* as true. At worst a researcher simply *fails to reject it*, perhaps due to insufficient statistical power in the study design. This misuse of a null hypothesis is a case of “heads we win, tails you lose,” in which the failure by LNT advocates to be able to reject a *proper* null of no effect (let alone benefit) below a threshold is attributed merely to insufficient data, whereas their opponents’ failure to reject an *improper* null of linearity is taken as evidence that the null is true.

##### EPA

The U.S. Environmental Protection Agency (EPA), through its director of the Radiation Protection Division, Jonathan D. Edwards, submitted a comment letter to the NRC in October 2015 urging the NRC to deny the petitions calling for an end to the use of LNT (Edwards 2015). The EPA based their position in part on the BEIR VII report and several epidemiological studies—Leuraud et al. (2015), Pearce et al. (2012), and Davis et al. (2015). The EPA notes that these studies “have shown increased risks of leukemia and other cancers at doses and dose rates below those which LNT skeptics have maintained are harmless—or even beneficial” (Edwards 2015). However, as we discuss elsewhere in the present article this is based on only a cursory reading of these studies at best, since an in-depth examination of them indicates that all these studies are flawed and their conclusions are unjustified.

Nevertheless, the EPA letter says,

Of all the agents demonstrated to be carcinogenic, the evidence for LNT is particularly strong for ionizing radiation. *Within limitations imposed by statistical power*, the available (and extensive) epidemiological data are *broadly consistent* with a linear dose–response for radiation cancer risk at moderate and low doses. Biophysical calculations and experiments demonstrate that a single track of ionizing radiation passing through a cell produces complex damage sites in DNA, unique to radiation, *the repair of which is error*-*prone*. Thus, no threshold for radiation-induced mutations is expected, and, *indeed, none has been observed*. (Edwards 2015; emphasis added)

These statements contain three glaring errors: first, as did Hall and Brenner (see above), it attributes the absence of evidence in favor of linearity in the low-dose range to lack of statistical power rather than to its (possible) nonexistence; second, it ignores any of the other mechanisms that come into play precisely when repair of DNA fails (listed above) and ignores the far greater damage done by normal metabolic processes through the production of reactive oxygen species (ROS) but which is also repaired to a greater extent as a result of low-dose radiation, thereby leaving fewer unrepaired DNA molecules than there would be in the absence of the low-dose radiation; and third, the claim that no threshold has been observed falsely denies the existence of the plethora of papers showing evidence of just such a threshold.

Indeed, even the data graphed by Davis et al. (2015) in the first figure of their cited paper clearly show the initial dip at low doses that is consistent with and suggestive of hormesis. Yet Davis and colleagues ignore this initial downward-sloping relationship, apparently regarding it as noise. Instead they attempt to fit to their data, by a priori assumption, both an upward-sloping straight line and a concave-upward quadratic curve with zero slope at the origin that then becomes positive but is nowhere downward sloping, exhibiting an inability or refusal to see the actual signal in front of their eyes—a refusal matched by the EPA's uncritical and cherry-picked reading of the scientific literature in this field. When a regulatory agency like the EPA endorses and employs false science it is no mere academic exercise.

Furthermore, the EPA has just issued a new warning about lung cancer ostensibly caused by breathing radon—a natural background source of radioactivity in the form of a gas that seeps up from the ground (EPA 2015). In their press release of November 10, 2015, they say, without any foundation in fact, “Exposure to radioactive radon gas is the second leading cause of lung cancer in America.”

The attempt to lower radon exposure has been shown to have the opposite effect at the dose rates encountered in homes—i.e., lowering radon exposure actually stands to *raise* lung cancer rates. It had been found in the 1800s that some European uranium miners suffered higher rates of lung cancer, and it was found, through controlled studies, that the primary cause was high levels of radon in the mines. Many mines, however, have far lower levels of radon, and many uranium mines, replete with radon, in the U.S. and Europe are used as health spas where people go to sit for hours and days breathing in the radon in order to palliate their arthritic pain and gain other healthful results. Somewhere between the high levels of radon found in some of the European mines and other mines and places, there must be a threshold above which the effect is harmful and below which it is healthful.

In the early 1990s a massive study was done by the late University of Pittsburgh Professor of Physics Bernard Cohen (1990, 1995, 2004, 2008, 2010), in which he attempted to measure the rate at which lung cancer increased due to increasing radon levels in homes. He examined some 1700 counties in the 48 contiguous United States, covering 90 % of the U.S. population. He found, much to his astonishment, that the higher the average radon level in homes within a county, the *lower* the lung cancer rate. He assumed that there must be some other variable that was confounding the measurement and reversing the expected finding, leading to this counterintuitive result. So he enlisted the help of a statistician, and together they analyzed the data for more than 500 combinations of possible confounding factors, including of course confounding by smoking. None of the possible confounders, either alone or in combination, explained the results. So Cohen was forced to conclude that it was the radon exposure itself that explained the inverse relationship with lung cancer, at least in the range of radon levels that he found in those homes.

Many attempts have been made to find the flaws in his study and in his conclusion, all of them successfully rebutted by Cohen (Puskin 2003, 2010; Heath et al. 2004; Puskin et al. 2004). After obtaining his unexpected result, Cohen sought the explanation in biology and discovered the existence of the hormetic effect, of which he had originally been ignorant. But Cohen was willing to switch to a new paradigm when the evidence demanded it.

When recently directly confronted by Stabin and Siegel (2015) with the proposition that LNT may grossly overestimate cancer risks associated with radon inhalation, Puskin and Pawel (2014) of the EPA responded that rejection of LNT is “indefensible when it comes to radon,” citing the study by Darby et al. (2015) as “proof” that LNT provides a reasonable estimate of risk at radon levels only slightly above the EPA action level. However, the Darby study is fatally flawed statistically, as we have previously pointed out (Siegel et al. 2014), since the authors merely assumed a linear association a priori between radon and lung cancer without any evidence of such. It is therefore no wonder that their result is consistent with LNT. Bayesian analyses using linear as well as other dose–response models indicated no evidence of such a linear dependence (Fornalski and Dobrzyński 2011; Dobrzyński et al. 2015). In fact, no association between radiation dose and increased lung cancer risk was demonstrated, even if Cohen’s data were excluded.

Yet the EPA continues to partner with and provide business to companies that seal basements and apply other methods to reduce the levels of radon, meanwhile possibly *increasing* the risk of lung cancer rather than decreasing it. This is yet another example of the way that ignoring biology leads to pervasive fear and adverse results—results for which no one is held accountable.

### Errors of Biological Omission

While there are those LNT advocates—like BEIR VII, Boice, Hall/Brenner, and Little (see below)—who admit that LNT cannot be proven in the low-dose range, due to the impractically large required sample sizes and the statistical noise resulting from smaller samples, there are other authors who believe that they have indeed detected, and proven, its reality in the low-dose range—i.e., that there is no threshold below which harm is absent. This contrary claim of detectability (and measurement) among many LNT advocates is exemplified particularly by two recent papers by Leuraud et al. and Richardson et al. (the same group of thirteen authors), writing for the International Agency for Research on Cancer (IARC), using data from the International Nuclear Workers Study (INWORKS) (Leuraud et al. 2015; Richardson et al. 2015).

Because biological considerations prevent the validity of such a conclusion, and because of the very widespread attention being showered on these studies by other authors as well as by regulatory and advisory agencies, we expend some effort here focusing on the erroneous (and contradictory) reasoning in the Leuraud and (to a lesser extent) Richardson papers.

It is not enough to demonstrate, as we have tried to do, that a certain approach—one that neglects and/or contradicts biological considerations in favor of sterile epidemiology—is *necessarily* flawed when that approach has enjoyed the *appearance* of success. When a study appears to have succeeded in accomplishing the impossible, it becomes necessary to examine it in detail in order to find and reveal its specific errors. Otherwise the issue is thrown into doubt.

It is also worth mentioning, to avoid conflating two different types of errors, that while the Leuraud paper stands as a prime example of the error of *biological* omission, at the same time it stands as an example of *commission* of multiple epidemiological, mathematical, and statistical errors as well, as we will illustrate. Following this analysis of Leuraud’s paper, we provide further justification for focusing such attention on this study and then describe several other prominent examples of biological omission containing equally false conclusions.

#### Leuraud and Colleagues

Leuraud et al. (2015), in their final paragraph and paraphrased in their abstract, characterize and promote their “conclusion” as follows (emphasis added): “In summary, this study provides *strong evidence* of an association between protracted low-dose radiation exposure and leukaemia mortality.” Since they explain that they are seeking the intensity of *risk* of leukemia due to chronic low-dose-rate radiation, for them the putative correlation signifies causation, even though in general the one does not necessarily imply the other.

The publicity surrounding the two IARC papers includes a podcast interview with the lead author of the first paper, Leuraud, by the journal that published it (*The Lancet Haematology* (TLH)). In it Leuraud reiterates her group’s “strong evidence.”^3^ The paper was also quickly publicized and praised in *Nature* (Abbott 2015), with its widespread distribution.

A few months later the same group of thirteen IARC authors, now with Richardson in the lead, published their second paper based on the same large INWORKS dataset, this time involving solid cancers rather than blood malignancies (Richardson et al. 2015). Their conclusion was essentially the same as in the first paper, albeit somewhat more modest: “The study provides a direct estimate of the association between protracted low dose exposure to ionising radiation and solid cancer mortality.” Leaving no doubt of their assumption of *causational* association, they end with their recommendation for mitigation: “Cancer risks that are associated with protracted radiation exposures can help strengthen the foundation for radiation protection standards.” If the search for causation were not their intent, radiation protection standards would be irrelevant.

Picking up on the intended *causal* finding in the Richardson paper, the World Health Organization (WHO) issued a press release (WHO 2015) saying: “This study strengthens the evidence of a causal relationship between solid cancers and exposure to low doses of ionizing radiation.”

And in the same issue of the *BMJ* (*British Medical Journal*) where the second paper is published, an editorial by Little (2015) cites both the Leuraud and Richardson papers approvingly and explicitly says of the latter: “This body of evidence does not suggest, and indeed is not statistically compatible with, any large ‘no risk’ threshold for dose, or any possible benefit (hormetic) effects.” In other words, Little agrees that the two treatments of the large INWORKS dataset (308,297 workers) by Leuraud and Richardson and colleagues rule out the existence of a threshold, and certainly rule out benefit below such a threshold.

Little also notes that “the excess solid cancer risks associated with radiation in this cohort are modest: for the average worker, the lifetime risk of cancer death is likely to be increased by about 0.1 % from a baseline risk of cancer death of about 25 %.” Consider this 0.1 % increase that Little is willing to pronounce “likely” in light of the 1–2 % increase that even BEIR VII questioned as detectable (quoted above). To pronounce the existence of an undetectable increase in cancer deaths as “likely” is characteristic of a reliance on assumption rather than on evidence, a reliance that is prevalent among LNT advocates.

Because such prestigious medical and scientific journals and major international agencies base, in part, their conclusions concerning radiation protection—a major public health issue—on such papers as these two, a detailed critique of them and, by implication, all others arriving at similar conclusions is rendered critically necessary.

As we demonstrate, their “conclusion” was based not only on a total eclipse of biological considerations, ones that would undermine the very premise of their study (the search for *risk* of cancer mortality *due to* low-dose radiation), but also—even within their biologically sterile approach—on *epidemiological* failure to exclude reasonable confounding influences, on unwarranted *mathematical* assumptions posing as inescapable, and on violation of *statistical* rules of inference.

We begin our critique of the Leuraud et al. study with the most egregious error and continue in descending order of importance.

##### Occupational Exposures Versus Natural Background and Other Radiation

The authors’ biggest error is the restriction of their cumulative radiation doses, on which they base their “strong” conclusion, to occupational exposures only. The mean dose rate for their 308,297 nuclear workers was “1.1 mGy/year, SD 2.6.” But there are many places in the world where the dose rate from natural background radiation is 10–100 or more times greater—as high as 260 mGy/year in Ramsar, Iran. Yet no higher incidences of cancer or mortality from presumed radiation-induced diseases have been found in these regions (Dobrzyński et al. 2015) or any other locations with high natural background.

The study categorizes subjects by their *cumulative* occupational exposures alone, which were no higher than 10 mGy over the entire 62-year study interval for three-quarters of their subjects. For comparison, natural background radiation for, say, a 50-year-old in a background region with 10 mGy/year, even leaving aside additional medical exposures (comparable to average natural background in the U.S., at around 3 mGy/year), would be 500 mGy—compared to 10 mGy occupational exposure. Thus, failure to account for natural background, or medical exposures, can lead to two workers with the same total cumulative dose being put in vastly different occupational dose-range bins. And conversely, two workers with vastly different total cumulative doses can be put in the same bin. So each bin contains workers with a wide dispersion of total cumulative exposures, rather than the relatively restricted range attributed to them by the authors through their estimates of occupational exposure only.

More importantly, *cumulative* doses, even correctly calculated, have no proven relationship to net outcome *when delivered at dose rates low enough to permit adequate time for repair*. Raabe (2015) concludes, in his review of internally accumulated radionuclides in both people (the radium dial painters; Rowland 1994, cited in Raabe’s paper) and experimental lab animals, “The cumulative radiation dose is neither an accurate nor an appropriate measure of cancer risk associated with protracted ionizing radiation exposure. At low average dose rates the long latency time required for radiation-induced cancer may exceed the natural lifespan yielding a lifespan virtual threshold for radiation-induced cancer….”

To imagine that cumulative dose produces a risk *regardless of how low the dose rate* is like imagining that a chef who cuts her or his fingers repeatedly over, say, a 10-year interval and loses a total of 5 L of blood over that decade, will die from exsanguination due to those serial finger cuts. Repair and healing save the day, and the chef. So does repair from radiation damage if given sufficient time. On the other hand, if a large enough dose of radiation is given over a very short time interval—short compared to repair or defense intervals—then indeed the person will die, just as the chef will die if she or he loses that volume of blood over minutes to hours (without transfusion).

##### LNT Model Assumed A Priori

In accord with the LNT paradigm, Leuraud et al. (2015) choose a priori, for the relationship between relative risk, RR, and cumulative dose, d, a linear model (straight line) that passes through their assumed “origin”: RR = 1 + βd, defining as the “origin” the point: d = 0, RR = 1 (*excess* relative risk ERR = 0). They then define the value d = 0 as zero *occupational* dose, neglecting all other sources of radiation, and the value RR = 1 as the cancer-mortality rate at d = 0, neglecting even conceptually (whether measurable or not) the cancer-mortality rate that would be found to occur at actual zero *total* dose, a significantly lower dose than zero *occupational* dose for any individual worker.

Since d = 0 corresponds to a wide dispersion of *actual* (rather than just occupational) cumulative radiation exposures, all of which are above zero with many of them *far* above zero, any line drawn though that arbitrarily defined “origin” is scientifically meaningless, let alone an index of actual correlation between cancer mortality and “cumulative dose.”

And if cumulative dose irrespective of dose rate were a relevant variable, mislabeling zero cumulative *occupational* dose as zero cumulative *total* dose in effect shifts all the curves to the left, thereby erasing much of the actual low-(cumulative) dose zone. Further compounding the error by mislabeling the response at zero dose as baseline—i.e., as zero effect or RR = 1 (ERR = 0)—in effect also shifts all the curves upward, thereby eliminating any possibility of RR < 1 (ERR < 0).

This effective combined shift of the relationship is common to many studies purporting to determine the slope of the dose–response relationship, while appearing to confirm LNT. However, the shift tends to erase the low-dose zone in which RR < 1 (ERR < 0) and thereby also *hides the region in which the dose*–*response relationship exhibits negative slopes*, leaving only that portion of the relationship that does indeed exhibit positive slopes, whether curved or straight. We return to this point below in our discussion of a recent National Cancer Institute (NCI) message to NRC advising rejection of the petitions calling for NRC to end the use of LNT and to acknowledge the evidence for hormesis.

In addition to the erroneous ascription of zero effect at zero occupational dose, the authors attempt to justify their selection of a straight line. They do so by comparing it to two alternative models, linear-quadratic and pure-quadratic, and find that the pure-quadratic model is actually mathematically better according to their chosen Akaike information criterion. However, they nevertheless select the straight line, admitting that it is for convenience, since the better quadratic model “did not substantially improve the model fit.” This is true, so far as it goes, but they consider no models other than these three. This vitiates their claim that it was their data that dictated the linear relationship rather than any assumption on their part. As they put it (as we discuss below), in their response to two pieces of correspondence objecting to the authors’ interpretation of their data and their conclusions (emphasis added):

*We did not simply assume that the data fit a linear model*…[rather] the trend in excess relative risk of leukaemia (excluding chronic lymphocytic leukaemia) with dose was well described by a linear function of cumulative dose, and…a higher order polynomial function of dose did not substantially improve the model fit. (Schubauer-Berigan et al. 2015)

**Fig. 1:**
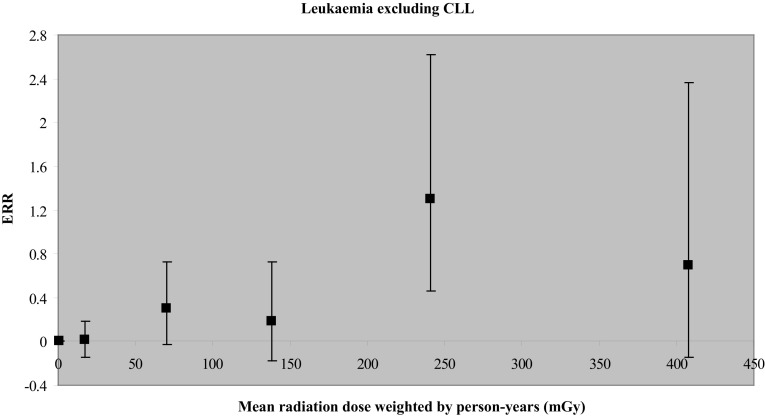
Graphic representation of Table A2 from Leuraud et al. (2015), showing the baseline point (at near zero dose) to be merely *assumed* to be the datum with the lowest value on the *y-axis* (without confidence interval), thereby disallowing the possibility that an actual *measurement* might result in a higher value than some or all of the other data points

However, the authors’ claim that the trend of ERR with cumulative occupational dose, for leukemia excluding CLL (chronic lymphocytic leukemia), is “well described by a linear function” is shown to be fallacious in the next section (in particular, see Fig. 1).

##### Absence of Statistical Significance Predominates, but is Ignored by the Authors and, Furthermore, is Obscured by Their Misleadingly Labeling the Data Merely as “Highly Imprecise”

The authors provide data for seven different blood and lymphoid malignancies. The data in their Table A2 (in their paper’s supplementary appendix) indicate that only one, chronic myeloid leukemia (CML), exhibits an ERR that appears statistically significantly (and positively) correlated with increasing long-term cumulative (but only occupational) radiation dose (again considering only a straight line). The slopes for all six of the other malignancies, even using the authors’ own data and their own choice of straight lines, *are consistent at least with the null of “no effect.”* They then supplement this paucity of confirmatory data by “finding” a positive linear relationship for an eighth, arbitrarily grouped, category, “leukaemia excluding CLL,” which combines three different diseases.

By artificially creating that new category, they imply that there are not one but two statistically significant positive associations—CML and “leukaemia excluding CLL.” But this eighth category achieves a statistically significantly positive slope solely due to the slope for CML—10.45 (90 % CI 4.48–19.65)—since AML (acute myeloid leukemia) and ALL (acute lymphoblastic leukemia) both have statistically *in*significant slopes when taken individually. It is permissible to achieve statistical significance for multiple small sample sizes by combining them, but only for samples with the same qualitative character and not for different diseases. Indeed the single excluded leukemia, CLL, actually has a *negative* point estimate for its slope, so including it would rob the slope for the category “all leukemias” of its statistical significance (though whether this would be noted, let alone admitted, by the authors is unknown). The authors justify their exclusion of CLL by acknowledging that CLL has no known relationship to radiation—their one (implicit) appeal to biological reality.

More revealing are the numbers in their Table A2. The RRs for only nine out of the 36 relevant cumulative dose categories (bins) are statistically significantly different from 1 (ERRs from 0); the other 27 are not. Furthermore, the trend across dose categories within each cancer is unsystematic, with these nine values scattered among the bins, contradicting the authors’ a priori assumption of linearity and positive correlation, but entirely ignored by them.

Perhaps the article’s most forceful, but untrue, claim is that “the RR of death caused by leukaemia excluding CLL by categories of cumulative dose showed a substantial risk for cumulative dose above 200 mGy” (Leuraud et al. 2015, pp. e278–279). However, a review of their Table A2 (the relevant portion is graphed by us in Fig. 1) reveals: (a) *only* the 200–300 mGy bin (average dose 241.2 mGy) has a value of ERR that is statistically significantly above 0 (RR > 1), i.e., with confidence interval excluding 0, but the still higher dose bin (>300 mGy, average dose 407.5 mGy) does not; (b) to put it another way, not one of the other dose bins, including the highest bin (also, after all, with “cumulative dose above 200 mGy”), shows statistical significance—which is to say, all the other bins, above and below 200 mGy, exhibit ERR values with negative lower confidence bounds (see Fig. 1); (c) this leaves the 200–300 mGy bin as the strongest contributor to the upward slope of an imposed straight line—reported by the authors to be 2.96 (our own calculation of that slope, 2.6, is in *rough* agreement, but only because of the constraint to go through the fictitious “origin”); (d) only 14 of the 531 deaths (2.6 %) determine this most influential 200–300 mGy data point; and (e) the data are heteroscedastic (have systemically varying CI widths), which generally precludes meaningful correlation.

Given the scattering of the data and the wide 90 % confidence interval error bars, any number of different shaped curves could be fitted to the data—either parametric (with a corresponding mathematical formula) or empirical (with no simple mathematical formula), not just a linear function or even linear-quadratic or pure-quadratic. So the authors’ claim that these data are “well described by a linear function” is misleading at best. It is further the case that the authors err in defining the putative zero dose (which is actually zero *occupational* dose and not zero *total* dose) as the baseline dose for which the response is assigned, *by definition*, the precise value RR = 1 (ERR = 0), without any confidence interval. Thus the origin in the graph is constructed in error and reflects both their failure to include the greater portion of actual exposures (from natural background and medical imaging) and their unfounded consequent *assumption* that their low-dose (as well as higher dose) region is characterized strictly by values of RR ≥ 1 (ERR ≥ 0), and strictly by positive slopes. But, as mentioned above, this misassignment of baseline response to zero *occupational* dose tends to erase that portion of the low-dose range with values of RR < 1 (ERR < 0), or with negative slopes, whereas including this portion would shift the dose–response curve back to the right and possibly downward where it belongs, potentially revealing the otherwise hidden hormetic portion of the curve (including a portion with negative slope), as well as the presence of a threshold dose. In contrast, the hormetic portion (along with its threshold) is indeed revealed in biological experiments, and even in many epidemiological studies. It is no wonder then that hormesis-obscuring practices lead to uncritical conclusions like that of Little (2015) in the quotation above from his editorial in the *BMJ*.

The authors describe their estimates of the six positive slopes (out of seven), and more relevantly those for the three (out of four) leukemias, as “highly imprecise” rather than the more revealing, and more accurate, “not statistically significantly different from no-effect.” Regardless, their conclusion that there is “*strong* evidence” (emphasis added) of positive associations between protracted low-dose radiation and leukemia is not warranted, even if we accept their arbitrary assumptions and invalid statistical procedures. That is, when an association is not statistically significant it cannot be said to constitute evidence at all for such an association, let along *strong* evidence—nor evidence for *risk* (a concept that necessarily implies causation, a point that we discuss further below).

##### Age as a Possible Confounder

Improbable as it might be that a positive slope for six out of seven categories is due solely to chance, it could still happen. But a more plausible explanation for this otherwise improbable outcome lies in one hidden confounder—age. The authors state they had stratified by age, but without revealing their findings, so we cannot check to see if there was sufficient evidence to reveal the confounding, or whether they simply overlooked it, as they did the absence of statistical significance.

If cumulative dose, particularly occupational, strongly correlates with age, and since most cancer mortality also strongly correlates with age, then cumulative occupational dose and cancer mortality would also strongly correlate, not because radiation causes cancer but because both are related to a common independent variable. Furthermore, age is more strongly correlated with occupational than with lifetime exposure due to the tremendous variation in natural background dose rates and relatively more, though by no means entirely, uniform occupational dose rates. Thus their failure to rule out this plausible confounder further undermines their conclusion.

##### The Use of Narrow Confidence Intervals

The authors note, “[b]ecause the objective of most contemporary radiation epidemiological studies is to investigate the potential for an *increased* cancer risk in relation to radiation exposure, one-sided p values and corresponding 90 % CIs are usually presented; we follow that convention here by reporting 90 % CIs” (Leuraud et al. 2015, p. 278; emphasis added). By looking only for *increased* cancer risk, this study, as do all similar studies, essentially ignores, as though operating in a parallel universe, voluminous published data that demonstrate *decreased* risk at low doses and dose rates, as well as the biological mechanisms that might explain such decreases (see above).

The a priori use of narrower CIs retracts the lower confidence limits toward, and possibly into, the positive range, making it more likely that the results become statistically significant. This outcome appears in the authors’ Table A5 for “leukaemia excluding CLL” in the stratification line “excluding UK,” where the lower 90 % CI for slope is 0.03. Using the more conventional 95 % this CI for slope would also include zero and fail to differentiate from “no effect,” or even protective effect. And, as noted, even with the narrower 90 % CIs, six out of the seven cancers in this study (and three out of four leukemias) still have statistically *non*-significant slopes.

Nevertheless, Leuraud et al. arrive at a conclusion that is not justified by their own data, or even their own analysis of it. This highlights an endemic problem for complex papers submitted to scientific journals, in which busy readers, lacking either the time or interest to read papers in their entirety, confine their reading to abstracts and conclusions, never suspecting that the conclusions may not be supported in the bodies of the papers.

### Why We Focus So Much Attention on the Paper by Leuraud et al.

One possible corrective to flawed articles and inadequate peer review lies in the letters of criticism submitted to journals that print such papers. But it is then incumbent on editors to encourage and facilitate the publishing of such critiques. We submitted to the publishing journal (TLH) a version of our foregoing critique of the Leuraud paper, primarily to expose it as an example of the error of *omission* of any appeal to biology and secondarily as an example of multiple errors of commission in its handling of epidemiology, mathematics, and statistics; but our submission was rejected primarily on the grounds that the journal was just about to publish two similar responses. However, those published correspondences (Doss 2015; Nagataki and Kasagi 2015) took a narrower approach. Indeed the only points addressed that overlapped with ours were the mention by Doss, in passing, of one instance of the raising of the lower confidence limit by the authors’ arbitrary narrowing of the CI—arbitrary but not uncommon in this sphere of biologically neglectful papers—and the mention in both items of the importance of including medical exposures.

In their response to Nagataki, Kasagi, and Doss, the authors actually divulged that the data for individual medical exposures were unavailable to them, but they nevertheless arrived at their conclusion—“strong” conclusion—even in the absence of such information, claiming without justification that it could not possibly affect their results (Schubauer-Berigan et al. 2015). They even attempted to justify their neglect of medical exposures, saying, “Individual information on radiation doses from medical procedures is unavailable in INWORKS, *as is the case for most occupational epidemiology studies*” (2015; emphasis added). They might as well have manufactured their cumulative exposure data out of whole cloth, particularly since both the neglected medical and natural background exposures are not trivial in comparison with their occupational data, but rather together they may outweigh it by orders of magnitude. And in their response (in the italicized portion of the quoted sentence above) they inadvertently lend support to our contention that the fallacious measure of cumulative radiation exposure is a common error within this entire class of epidemiology studies.

Committing perhaps a worse transgression, the authors failed to even acknowledge the existence of some references that were provided by one of the correspondences (Nagataki and Kasagi 2015) that demonstrate the opposite of their conclusion. To obscure rather than elucidate their many errors and their false conclusion, the authors offered the following attempt at shielding themselves against further criticism: “In summary, the INWORKS study (like most observational epidemiology studies) has limitations, which we believe have been adequately described in our Article, and which, in our opinion, do not greatly affect its conclusions” (Schubauer-Berigan et al. 2015).

As we demonstrate, the “limitations” of their study are by no means “adequately described” by the authors—particularly their failure to appeal to biological considerations. But the “limitations” are far less significant than their multiple epidemiological, mathematical, statistical, and data-handling *errors*. And, as we further demonstrate, these limitations, and even more importantly their errors, not only do indeed “greatly affect” their conclusions, but they entirely nullify them.

Thus this paper—in accord with the confessions by certain past and present journal editors, quoted above—reveals an inadequacy of peer review at least for papers based on the LNT paradigm that goes along with a paradigm blindness. And as we have mentioned, this paper, along with many similar studies, receive laudatory attention and citations in numerous journals and popular media. Meanwhile papers that demonstrate the falsity of LNT—such as those by Cohen (described above) or Sponsler and Cameron (2005), who found that nuclear shipyard workers experience lower rates of cancer and enjoy greater longevity than their fellow shipyard workers whose work is remote from the nuclear reactors (a control group chosen to eliminate the healthy worker effect—a frequent assumption proffered to explain studies demonstrating hormesis) do not enjoy such lackadaisical review. Instead they are the subject of concerted, though inaccurate, efforts at refutation (Boice 2001; Puskin 2003, 2010; Heath et al. 2004; Puskin et al. 2004).

We assuredly do not advocate that such studies also be exempt from piercing peer review, or deny that such studies may also require refutation, if warranted, but this double standard is indicative of a severe problem, at least in radiation science.

This double standard, with its faulty peer review process, partially explains the state of affairs described above by Horton, Angell, and Ioannidis, with some half of published science papers containing nontrivial errors. The Leuraud paper, along with its companion paper by Richardson et al. (the same group of thirteen authors), constitutes yet another contribution—this time major and already much cited—to an entire class of invalid papers in scientific journals, with all the concomitant likely damaging results to millions of people, mentioned in our introductory comments above and revisited in more detail in our penultimate section below.

Prominent among the writings that uncritically cite, among other similarly flawed studies, the IARC INWORKS studies by Leuraud and Richardson et al., is a recent message sent to the NRC in the name of the Radiation Epidemiology Branch of the NCI of the National Institutes of Health (NIH). NCI cites these papers as support for their advice to the NRC that they reject the three petitions calling for an end to the use of LNT (Berrington de González et al. 2015).

In addition to their uncritical reliance on such flawed studies, the NCI authors commit the error of invoking, in typical fashion, the lack of statistical power as their explanation for the non-detectability of the presumed carcinogenic effects of low-dose radiation in many other studies. Again, typically, they never question whether such detrimental effects actually obtain. On the contrary, at one point they claim that in one cited study “a statistically significant positive association of solid cancer with radiation dose was found, which certainly does not support hormesis as the petitioners claim.” Yet, as we have shown in point 2 of our critique of the Leuraud study above, by discounting much of the actual radiation received, and thereby shifting the dose–response relationship up and to the left, that portion of the actual low-dose region in which the slope and the ERR are both negative is erased. This shift produces the illusion that the dose–response relationship has everywhere a positive slope and that therefore there is no hormetic zone.

We provide our critique of the Leuraud study in such detail in order to suggest ways that similar critiques should be performed of the other studies cited by NCI that circularly conclude that LNT is valid.

As we stated above, paradigm blindness often causes those in its grip to regard signal as though it were noise, and vice versa. This reversal is facilitated by the brevity of correspondences in response to erroneous papers. In contrast, a longer and more detailed critique, such as this one, has a better chance of being noticed as signal, by both authors and readers, than the shorter responses, which can be brushed off as part of the din.

But more important yet is the fact that the last court of appeal for the validity of scientific studies lies with the readership of journals, particularly when erroneous studies are passed by peer reviewers and editors. Publication of a fully detailed critique affords the scientific community a better chance to judge both sides of an issue.

Finally, despite Leuraud et al.’s failure to take into account biological considerations at all and instead rely solely on statistical and mathematical relationships, not to mention their commission of numerous errors, their paper is having, and will undoubtedly continue to have, a tremendous impact. Unfortunately, in this case it is a wholly unwarranted negative impact. This entire class of papers must be held to account because of the enormous harm for which their conclusions can be responsible. If these studies are improperly designed with respect to data collection and/or analyses, their conclusions will be erroneous and unless revealed to be false, are likely to be used to support policies and regulations that are highly damaging and often deadly to thousands and thousands of people. Those of us who defend science and seek to further its influence have an immense responsibility to arrive at the truth and not to further purvey erroneous and pseudo-academic musings posing as scientific studies.

## Other Examples of the Error of Biological Omission

Here are a few other examples of the error of biological omission in the use of LNT that we mention without analyzing in detail to give some idea of the pervasiveness of this sterile approach.

### Spycher et al.

Spycher et al. (2015a), in a study titled “Background Ionizing Radiation and the Risk of Childhood Cancer: A Census-Based Nationwide Cohort Study,” claim to have detected and measured a linearly proportional increasing cancer rate in Swiss children from terrestrial gamma and cosmic radiation in the range of about 0.9–1.8 mSv/year, which turns out to be (though the authors ignore this fact) slightly less than the average total natural background exposure of 2 mSv/year in the world from these sources plus radon, the major source of terrestrial background radiation. In fact, as mentioned previously, there are places in the world where the natural background radiation dose rate is two orders of magnitude higher.

Importantly, the background exposure rates were based not on actual measurements at children’s homes but on a geographic model. Furthermore, the authors ignored the important potential dose contribution of radon. The authors even noted they could not “exclude biases due to inaccurate exposure measurement,” but this did not prevent them from concluding that, “It is plausible that the observed associations between background radiation and childhood cancer reflect a causal relationship” (Spycher et al. 2015a, p. 627). Thus even if we were to accept that the radiation were the cause of childhood cancers, all their background exposure rates are inaccurate and incomplete and any attempt at correlating these rates with *any* effects, let alone increased cancer rates, is fallacious.

In our published comment (Siegel et al. 2015b) criticizing this claim we showed that it was based on the a priori assumption of LNT and that, even if the correlation were valid, their claim of cancer *causation* by radiation was unwarranted. In their response to our comment, despite specifically having opined, in the sentence quoted above, that causation was a plausible conclusion, they denied any intention to imply causation, reciting the general denial that correlation does not necessarily imply causation (Spycher et al. 2015b). In particular, they said,

Siegel and colleagues object to our use of the word “risk” on the basis that it implies a causal relationship. This is not so. In epidemiology, risk is simply the probability of developing the disease. Comparing risks across exposure strata is a natural way of assessing associations in a cohort study and does not imply causality. Our conclusions regarding causality are, in fact, very cautious.

But in their paper, in addition to their expressed claim of the plausibility that background radiation *causes* childhood cancer (quoted above), the opening sentences of their introduction—citing as sources the United Nations Scientific Committee on the Effects of Atomic Radiation (UNSCEAR) and a few other authors—read:

Ionizing radiation is a known *risk factor* for cancer. For a given radiation dose, children are at a greater risk than adults. Ionizing radiation is the only established environmental risk factor for childhood leukemia and tumors of the central nervous system (CNS), the two most common tumor types in childhood. (Spycher et al. 2015a, p. 622; emphasis added)

There is a difference between a risk *factor* and a risk *marker*. The latter is generally a feature that shares a common cause with the disease in question and is therefore not itself a contributing cause, while the former implies a *causal* role in the development of the disease. Indeed the very word “risk” implies causation. Thus the authors, while appealing to LNT in their research and paper, are not forthcoming about the a priori nature of that assumption, which they employ in place of any appeal to biological considerations when calculating cancer risk in the very low-dose-rate range. Instead they appeal to other similar biologically empty “findings,” labeling radiation, regardless of dose, as “known” to be, and “established” as, a risk *factor* for malignancy in children.

Incidentally, Spycher et al. (2015b), in their response to our critical comment, also said,

Childhood cancer is rare, and we are not dealing with deaths at “alarming rates.” In the whole of Switzerland, there are about 200 new cases per year, of whom more than 80 % survive (SCCR 2015). Only a small proportion of the population is living in highly exposed areas. The attributable fraction, assuming a causal relationship, is therefore small. Public health action is better targeted toward modifiable environmental factors leading to larger numbers of deaths from several causes, such as exposure to radon, air pollution, and secondhand tobacco smoke.

Thus even as they defended their study and conclusions, they admitted that their finding should not impact policy decisions, and that public health action would be better targeted elsewhere. One has to wonder, what then was the motivation for their study in the first place, and for its funding.

And finally, reversing the charges, Spycher et al. ended their response with,

It seems to us that the “Scientists for Accurate Radiation Information” a priori exclude the possibility that low-dose radiation could increase the risk of cancer. They will therefore not accept studies that challenge their foregone conclusion. (Spycher et al. 2015b)

Thus they ignored the fact that the existence of a threshold and the reality of hormesis rest on solid evidence while LNT rests precariously on a sandpile of assumption, instead charging that it was our response rather than their study that reflected a priori bias. (Scientists for Accurate Radiation Information, or SARI (www.radiationeffects.org), is an international organization of which the seventeen authors who disputed Spycher et al.’s contentions are members.)

### Kendall et al.

Again, examining natural background radiation, Kendall et al. (2013) in Great Britain performed a large record-based case–control study suggesting an excess risk of childhood leukemia associated with natural background radiation exposure. There were approximately 27,000 cases born and diagnosed in Great Britain and approximately 37,000 matched cancer-free controls. The authors reported that, “There was 12 % excess relative risk (95 % CI 3–22; two-sided p = 0.01) of childhood leukaemia per millisievert of cumulative red bone marrow dose from gamma radiation” (Kendall et al. 2013). They concluded that the statistically significant leukemia risk reported in this reasonably powered study (~50 %) supported the extrapolation of high-dose-rate risk models to protracted exposures at natural background (low-dose-rate) exposure levels, which they explicitly regarded as causal.

However, according to an UNSCEAR report, this study “should be interpreted with caution because of the large uncertainties associated with using an ecological measure of dose” (UNSCEAR 2013, p. 77). Radiation doses in this study were based on estimated mean exposure levels for the county district in which the mother resided at the child’s birth. Thus, there was a huge uncertainty associated with these assigned radiation doses as individual dosimetry was not performed. Further, although the authors concluded that substantial bias was unlikely, they specifically admitted that “The study has no information on potential confounders other than measures of socioeconomic status, and the causes of the majority of cases of childhood leukaemia remain unknown” (Kendall et al. 2013). So the authors contradicted themselves with respect to their assertion that the noted association was causal.

Again, this paper neglected any mention of biological considerations and arrived at an epidemiological conclusion resting on mathematical assumptions similar to those discussed above.

### Pearce et al. and Mathews et al.

These two epidemiological studies suggested an increased cancer risk at low doses associated with pediatric CT scans. Pearce et al. (2012) performed a cohort study of almost 180,000 juveniles less than 22 years of age in Great Britain. An increased incidence of leukemia and brain tumors was reported. The authors concluded that

Use of CT scans in children to deliver cumulative doses of about 50 mGy might almost triple the risk of leukaemia and doses of about 60 mGy might triple the risk of brain cancer. Because these cancers are relatively rare, the cumulative absolute risks are small: in the 10 years after the first scan for patients younger than 10 years, one excess case of leukaemia and one excess case of brain tumour per 10,000 head CT scans is estimated to occur. (Pearce et al. 2012)

Again, this study purported to attribute causation (“risk”) to the radiation from the CT scans for childhood malignancies, in this case leukemias and brain cancers.

Mathews et al. (2013) performed a cohort study of 11 million juveniles in Australia—680,000 were exposed and all study participants were less than 20 years of age. An increased cancer incidence (of all types) was reported and the authors stated that “The increased incidence of cancer after CT scan exposure in this cohort was mostly due to irradiation” (Mathews et al. 2013). These authors at least did not deny, but rather explicitly stated, their conclusion that the radiation from the CT scans was the cause of the increase in cancer rates.

### Journy et al. and Krille et al.

A recent large-scale cohort study in France involving more than 67,000 children (Journy et al. 2015) addressed the question in the article’s title: “Are the studies on cancer risk from CT scans biased by indication?” Adjustment for *cancer*-*predisposing factors* reduced the excess risk estimates related to cumulative doses from CT scans such that no significant excess risk was observed in relation to CT exposures. It was concluded that the indication for the CT examinations should be considered to avoid overestimation of the cancer risks associated with CT scans. However, since the mean duration of follow-up in this study was only four years—too short to provide any conclusive results about radiation-induced risks—this study by itself has admittedly not provided sufficient evidence to invalidate the risk predictions extrapolated from studies at high doses under the LNT assumption. Studies extending the follow-up period are ongoing.

In a separate recent German cohort study examining the risk of cancer incidence after exposure to ionizing radiation from CT, Krille et al. (2015) noted that “Despite careful examination of the medical information, confounding by indication or reverse causation [i.e., an already present cancer in a child, or the presence of predisposing illness, gives rise to the use of CT scans, rather than the other way around] cannot be ruled out completely and may explain parts of the excess” cancer cases observed—something that neither Pearce et al. nor Mathews et al. even considered. Krille et al. (2015) continue, “Furthermore, the CT exposure [of the children studied] may have been underestimated as only data from the participating clinics were available. This should also be taken into account when interpreting risk estimates.”

The caution with which authors like Journy et al. and Krille et al. merely question such findings rather than refute them on the basis of reverse causation is in part a reflection of the intimidating political dominance by the LNT paradigm in both the scientific literature and in widespread regulatory policies.

## The Dominating Paradigm of LNT is Without Scientific Substance but Wields Tremendous Influence

The Pearce and Mathews studies do not provide evidence that CT doses are causally associated with cancer in children. Not only have questions of reverse causation and inaccurate dosimetry been raised to throw doubt on their claims, but significant concerns have been raised about the quantitative risk estimates in these studies (UNSCEAR 2013; Walsh et al. 2014). Since these two pediatric CT studies do not provide evidence that low doses are causally associated with cancers in children, direct estimation of the health impact of CT radiation exposure based on them remains out of reach. Rather, based on biological considerations, it is possible to conclude that any negative impact of the associated radiation exposure is not only undetectable but is nonexistent.

Again, the failure to appeal to biological considerations, and a fealty to the LNT paradigm, lead authors to overlook otherwise obvious confounding conditions and arrive at unwarranted conclusions. All these studies lack accurate dosimetry and employ circular reasoning, but every one of them that appears in the literature is touted as yet more confirmation of LNT. When one begins with an a priori assumption it is no mystery why the conclusion may be taken to confirm it. As mentioned above, busy scientists and physicians rarely have the time to read with an adequately critical attitude, leaving the sheer volume of such studies to stand in place of scientific worth or validity.

Agencies and organizations like the BEIR committee, the NRC, the EPA, and IARC, as well as individuals like Hall, Brenner, Boice, Little, and the authors of studies like those we have reviewed here, have developed a longstanding vested interest in preserving the LNT fiction (Calabrese 2015). Their continued support for LNT year after year undergirds their ongoing funding and reputations, which in turn are in the hands of politically powerful governmental and private funding agencies along with publicizing media that bring the scientific issues and positions to public attention and thus reinforce and prolong the life of scientifically discredited paradigms. This societally rooted conflict of scientific interests creates an obstacle to serious examination of the biological realities. The resulting mass radiophobia joins hands with the LNT paradigm to produce extremely harmful consequences. We end our essay with an examination of three key examples of such consequences.

## Adherence to LNT Produces Mass Radiophobia, Which is Very Bad for Your Health

The LNT paradigm, used as a scientific icon and as a guidance for regulatory policy, promotes radiophobia in masses of people, as well as in governments, around the world through its percolation upward into popular culture, media, and mass movements (Sacks and Meyerson 2015). Mass fear is one of the easiest to inculcate and manipulate for self-serving and harmful ends and one of the most difficult emotions to overcome.

The harmful effects come in several forms, including forced evacuations at sites of nuclear power plant accidents, widespread refusals by people to avail themselves of needed radiological imaging studies, and an all-too-common (though far from universal) aversion to nuclear energy to replace fossil fuels. We discuss these in order of the most immediate to the more delayed impacts.

### Forced Evacuations of Hundreds of Thousands

It has been observed that overestimating radiation risks based on LNT may have worse outcomes than underestimating them (Siegel and Welsh 2015). For example, the fear produced by the belief that LNT is true—i.e., that all radiation is harmful no matter how low the dose or dose rate—with its erroneously extrapolated and unsupportable threats to public health, resulted in unnecessary loss of life following the Fukushima and Chernobyl nuclear accidents, due to traumatic forced evacuations and fear-driven suicides, and in the case of Chernobyl, panic-inspired abortions. The Japanese government’s mandatory relocations of some 150,000 people from Fukushima after the 2011 earthquake and tsunami has created mayhem, as official figures indicate more than 1600 deaths were a direct result of the forced evacuations, and the evacuation orders are still in effect after four years, as reported in *The Japan Times* (2015). Initially, when radiation doses were unknown, the evacuations may have been justified, but when the involved doses of 12–25 mGy radiation exposure in the most affected regions and 1–10 mGy to all other residents, projected for the first year, became known—as they did relatively quickly—the evacuees should have been given the “all clear” and allowed to return home (Shimura et al. 2015). These doses over the course of a year, as mentioned above, are, after all, well below natural background radiation exposures from ground and sky in other parts of the world—where it is as high as 260 mGy per year.

According to, among many other sources, the United Nations Information Service (2013):

Radiation exposure following the nuclear accident at Fukushima-Daiichi did not cause any immediate health effects. [Furthermore, it] is unlikely to be able to attribute any health effects in the future among the general public and the vast majority of workers.

Following the Fukushima accident, the International Commission on Radiological Protection (ICRP), a non-governmental independent scientific organization, convened Task Group 84 to collate in a memorandum the lessons learned. According to the Task Group (González et al. 2013, p. 510),

Following exposure to low radiation doses below about 100 mSv an increase of cancer has not been convincingly or consistently observed in epidemiological or experimental studies and will probably never be observed because of overwhelming statistical and biasing factors.

In sum, theoretical cancer deaths after low-dose radiation exposure situations are obtained by inappropriate calculations based on the LNT model and misuse of the collective dose concept [see fourth paragraph in our Introduction above]. Any effects—if they occur at all—will be so small that they would fall within the “noise” (scatter) of the “spontaneous” cancer of unexposed people.

Thus, while these official statements equivocate slightly, and while estimates of increased radiation-induced cancer risks at low doses are often predicted, these risks are mathematical fictions based on the demonstrably false LNT hypothesis and its associated theoretical model. The absence of harm to public health, with actual benefit, is the evidence-based predictable outcome.

The situation in the three countries surrounding Chernobyl (Ukraine, Belarus, and Russia) is far worse, with the mental health impact of the 1986 nuclear plant accident—including increased alcoholism and stress-related heart attacks and strokes—being the largest public health problem resulting from the accident (WHO 2006; Siegel and Pennington 2015). Voluntary abortions and suicide rates increased in Western and Northern Europe due solely to LNT-driven radiophobia, despite the absence of data suggesting harmful genetic effects or increased solid cancers or leukemia, or any other non-malignant disorders due to low-dose radiation.

There were 134 emergency responders with clinically confirmed diagnoses of acute radiation syndrome (ARS). According to WHO, “Among these 134 emergency workers, 28 persons died in 1986 due to ARS, and 19 more died in 1987–2004 for different reasons” (WHO 2006, p. 99). The 28 who died were exposed to the greatest radiation doses during their ten-day efforts to extinguish the fire.

Chernobyl’s longer-term cleanup personnel have also been studied. The Estonian cleanup workers, for example, received an average radiation dose of approximately 100 mGy (a low dose, but certainly higher than the average dose received by other Estonian males). However, even though this dose is equivalent to that received from approximately ten CT scans, the study report concludes the following (Rahu et al. 2013):

…after a quarter century follow-up of the Estonian cohort…there is an increased risk of alcohol-related cancers and of suicide. No definite indication of health effects directly attributable to radiation exposure was found.

Still, even in the face of all the evidence against it, many, including the EPA, claim that the LNT model is conservative (errs on the safe side) and that any derived regulation or policy will be protective (Puskin 2009). The opposite, however, is the reality, yet no one is held accountable for the many resulting deaths so long as the LNT fiction holds sway, thereby shielding its proponents.

### Refusal of Radiological Imaging Studies

The LNT model underlies the fear that dissuades many physicians from using appropriate and adequate imaging techniques and discourages many in the public from getting proper and needed imaging. Any discussion of risks related to radiation dose from medical imaging procedures must be accompanied by acknowledgment of the benefits of the procedures (Balter et al. 2011; Cohen 2012). Radiation exposure from medical imaging is considered by many physicians and patients to be the only risk when accurate diagnoses of internal conditions are called for. But the more significant and actual risks associated with invasive exploratory surgical procedures that were necessitated prior to the invention of CT—and that continue to be necessitated by current physician and/or patient/parent refusals of CT scans, or misdiagnoses in the absence of, or underexposed and therefore nondiagnostic, CT imaging—are generally ignored in both the scientific literature and the popular media. The LNT model and the philosophy behind it are more concerned with the extremely small number of future, and only hypothetically (and erroneously) predicted, cancer occurrences and deaths attributed to radiation exposure than with the much larger numbers of actual deaths that are certain to occur without imaging. It is accepted radiological practice then to fall back on justifying medically indicated imaging procedures on the basis of favorable risk/benefit calculations, implicitly resting the presumed existence of risk on the foundation of LNT.

Medical imaging studies, including chest X-rays and CT scans—which expose the patient to radiation exposures on the order of 0.1 and 10 mGy, respectively—may be associated with a “negative” risk, i.e., a protective response (Scott 2008). The use of both radiological and nuclear medicine imaging has increased dramatically over the past 20–30 years, but there is considerable evidence of the effectiveness of these procedures in reducing morbidity and increasing average longevity. Since cumulative public radiation exposure has grown (most notably as a result of increased CT imaging)—along with fears that this additional radiation dose may be associated with radiation-induced cancer risk and genetic risk to future populations—policies and procedures have come into being that seek to minimize even further these putative low-dose radiation-induced risks. They are premised on the LNT model-driven assumption that such risks are real, an assumption that we have shown is not supported by either historical or contemporary experimental or (validly obtained) observational (epidemiological) data. Any approach touting the “known” cancer risks due to low-dose/dose-rate radiation exposure from radiological imaging procedures should be vigorously challenged, because it serves to alarm and often harm, rather than educate.

It is of course important to eliminate clinically *unwarranted* radiological imaging studies—as is true for any medical procedure whatsoever (Siegel and Stabin 2014)—but for reasons other than radiation exposure. While it equivocates on the nonexistence of harm at low doses, a statement by the American Association of Physicists in Medicine (AAPM 2011) says,

Risks of medical imaging at effective doses below 50 mSv for single procedures or 100 mSv for multiple procedures over short time periods are too low to be detectable and may be nonexistent. Predictions of hypothetical cancer incidence and deaths in patient populations exposed to such low doses are highly speculative and should be discouraged. These predictions are harmful because they lead to sensationalistic articles in the public media that cause some patients and parents to refuse medical imaging procedures, placing them at substantial risk by not receiving the clinical benefits of the prescribed procedures.

The class of papers purporting to demonstrate the cancer-causing effect of CT scans, as discussed above, leads to the call for lowering exposures to a level that is as low as is reasonably achievable (ALARA) and, via the so-called Image Gently campaign, and its adult counterpart the Image Wisely campaign, reducing CT exposures to children and adults, respectively.

As a result of these twin campaigns there has been a trend toward the use of too little radiation for the resulting CT scans to be diagnostic, leaving the interpreting radiologists uncertain of their findings and weakening the ability of the ordering physicians to help their patients (Boutis et al. 2013). However, when properly performed, CT scans often either strengthen confidence in prior diagnoses, leading to better treatments, or cause the managing physician to change from one diagnosis to a more accurate one (Pandharipande et al. 2015).

Those responsible for LNT- and ALARA-based recommendations and practices believe they are erring on the safe side by limiting radiation exposure, yet because of the claimed risk there are many patients and/or parents who refuse needed CT scans. Such patients and parents needlessly suffer solely because of unjustifiable alarmism nourished by the LNT paradigm (Pearce et al. 2012; Boutis et al. 2013; Medical Press 2015; Parker et al. 2015).

On a less intense, but still fear-inducing, level, there are papers and statements that correctly assert that there is no evidence that CT scans increase the risk of cancer, in children or adults. Yet they contradictorily advocate the use of lower doses of radiation for needed CT scans as a “prudent” approach, thereby conflating the actual prudence of confining any medical procedures to those that are clinically indicated with the false prudence of limiting radiation exposures in the context of clinically indicated imaging (McCollough et al. 2015). Thus, apparently afraid to wander too far out on a limb in the face of the dominating and intimidating, but erroneous, LNT paradigm, they undermine their own messages of reassurance, leaving patients and/or their parents confused as to whether there is risk or not.

Falsely vilifying imaging in the absence of actual confirmatory data and in apparent ignorance, or at least neglect, of much contrary observational and experimental data, and particularly without regard to the risks of its alternative surgical or other less accurate diagnostic approaches, can be deadly.

### Aversion to Nuclear Energy

In place of, and to avoid investigating, nuclear energy, laudatory attention is often focused on the so-called “renewable” sources, wind and solar. But this attraction rests on a one-sided failure to take into account the nature of the devices and their constituent materials needed to convert into electricity these otherwise plentiful, clean, and sustainable forms of energy. It is not the energy sources that matter so much as the nature of the devices required for their conversion to electricity and other useful forms. The all-too-common willingness to accept uncritically the proclamations from nuclear opponents and wind-and-solar proponents rests firmly on the foundation of radiophobia, in turn inspired by the LNT paradigm, as its often unrecognized subtext.

It would be much too far afield to examine here the pros and cons of “renewables” versus nuclear energy, which is covered in depth elsewhere (Sacks and Meyerson 2012, 2015). But suffice it to say that radiophobia immensely distorts and inhibits this examination and debate, and makes enemies out of would-be allies. This often-reflexive fear reaction inhibits many from even seriously investigating the use of nuclear energy.

## Conclusion

LNT-based radiophobia fuels needless evacuations, inspires avoidance of life-saving medical procedures, and promotes nuclear fear. Considerations of the basic sciences of biology, physics, chemistry, and other natural sciences should be either the source or the final arbiter of scientific hypotheses about ionizing radiation, and not sterile epidemiological studies, designed to yield mathematically convenient relationships, that ignore the manifold findings of those basic sciences and rest their conclusions on circular reasoning. Failure to take proven biological reality into account leads to counterproductive statistical exercises, sometimes fraught with numerous errors, that carry the misleading appearance of erudition through mathematical complexity. These studies are not benign; they do not err on the safe side; and they have deadly consequences.

This unscientific practice must end, for the sake of much of humanity.

## Commentary on Sacks, Meyerson, and Siegel’s “Epidemiology Without Biology” by Inge Schmitz-Feuerhake^4^

The basic assumption of the authors is wrong: there is no LNT hypothesis in radiation biology. Rather, the hypothesis is that “stochastic” effects exist. That means that one single quantum of radiation is able to produce a mutation in the genetic material of a cell. This altered single cell may become the origin of an uncontrolled proliferation or, in the case of a sexual cell, of a hereditary effect. The LNT dose-dependency is just a practical approximation for radiation protection. For example, Sacks and colleagues cite Ozasa et al. (2012), whose report on their investigations of solid cancer mortality in atomic bomb survivors states that assuming dose-proportionality for the effect gives the best fit in the analysis of the data in the region 0–3 Gy. But they also find that “the dose–response slope was nominally higher at doses below 0.1 Gy than it was overall” (2012, p. 238). Their formal dose-threshold analysis indicated no threshold.

Hazelton et al. (2006) studied lung cancer in about 190,000 workers of the Canadian National Dose Registry and found a strict inverse dose-rate effect for low LET irradiation, which means that the excess relative risk (ERR) generally increases with protraction of a given dose, an observation that was often reported after internal alpha exposure. This indicates that any adaptive response—which is certainly dependent on dose-rate—will fail in cases of very low dose-rate.

The stochastic effect is a rare event after low-dose exposure, and, therefore, no scientist claims that “*all* acute exposures to ionizing radiation are harmful…*regardless of how low* the dose,” as Sacks et al. state (emphasis in original). The effect appears because of misrepair or failure of the immunological or adaptive response or a combination of these factors. It is hardly biological understanding to believe that every instance of primary radiation damage will be completely compensated or lead to precautionary reactions in each individual, whether healthy, ill, predisposed, or temporarily distorted.

The article regards only cancer and leaves out the hereditary risk. In the case of a mutation in a haploid sperm that leads to a mutation in the zygote and the descendant, several or all of the mechanisms of repair listed in the authors’ section on adaptive responses will not work. De novo mutations from Chernobyl fallout were not only shown in animals (e.g., by the group Moller/Mousseau (Moller et al. 2005)) but also in children of Chernobyl parents (Weinberg et al. 2001; Dubrova 2003). The Belarus central registry for congenital anomalies shows rising rates after Chernobyl and severe increases in regions of high contamination in comparison to those of lower contamination. Some of them are confirmed de novo mutations (Lazjuk et al. 1997). Another example is Down syndrome, which increased in several contaminated regions after Chernobyl (Sperling et al. 2012). Thus, former findings in Kerala or high background regions in China or after diagnostic X-raying were confirmed. Evidently, nondisjunction induced by radiation will not be repaired or eliminated.

And what about the developing system? The findings of Alice Stewart in the 1950s about leukemia and other cancers in children exposed in utero to diagnostic X-rays were denied for decades, but finally accepted by the officially charged committees because of numerous confirmations.

An improved protection against stochastic effects at low doses is necessary because, although rare, they may be fatal or dramatic as, for example, cancer in childhood, severe malformation, mental retardation, or may affect further generations. The article offers no convincing new argument against the existence of stochastic effects.

“Radiophobia” was invented by pro-nuclear colleagues in order to explain diseases after Chernobyl. But nobody has proven that phobia causes mutations and cancer. Why not assume that there is adaptive response, because phobia has probably also existed since the beginning of mankind?

## Some Comments on Sacks, Meyerson, and Siegel’s “Epidemiology Without Biology” by Christopher Busby^5^

The authors employ a philosophical argument as a platform from which to launch an attack on the current LNT approach to radiation risk, one which is enshrined in law. Philosophical arguments are welcome in science and are quite rare. I welcome this attempt to unpick the origins of radiation risk and its accepted components. I will take issue with this philosophical argument and accept most of the (cherry-picked) evidence as reasonably accurate. The issue of the correct dose–response relationship is an important one in the area of public health. The authors conclude that the LNT model is incorrect and that it should be replaced with a threshold model; they espouse the concept of hormesis whereby a small dose of ionizing radiation up-regulates cellular defenses and DNA repair efficiencies which effectively protect against genetic lesions that lead to cancer and other health effects in those exposed or presumably, through the same mechanisms, their progeny. In their worldview, small doses of radiation are “good for you.”

The concept of hormesis is based upon a failure of scientific philosophy. It is a truly dangerous thesis because adopting the measures that it suggests will result in a serious increase in illness and genetic damage in members of the public and workers who are currently protected under a regime which itself fails to take into account evidence (including that presented by the advocates of hormesis). The article is an interesting example of this failure in the very areas of natural philosophy that the authors are addressing. The authors have themselves done all the things that they accuse the regulatory system of having done. It is easy to capture their argument, and I will do so. Essentially, what they do is choose an interpretation of their selection of the observations but fail to take their analysis far enough.

Their title, “Epidemiology Without Biology” is a good one: but (perhaps because of ignorance, or reductionism) they have failed to examine the full biological picture. They have two basic false assumptions:

1. That the various studies that appear to show a sparing of effect at low external dose, do not begin the trend at the origin, the point (0,0) zero dose, but at background levels, maybe 2 mSv most of which is internal radon.
2. They fail to understand that the concept of dose itself is not valid for internal exposures, which convey the predominant risks from radiation. This error is also present in all the nuclear worker studies that reference their results to the low-dose group rather than to a proper control group or the national database. The latest example of this questionable epidemiology is the INWORKS nuclear workers studies (Richardson et al. 2015). This epidemiological failure problem began with the Japanese LSS studies where, following the finding that there were low levels of cancer in the control group, the Not-in-City (NIC) group (the externally unexposed controls) were abandoned in favor of referencing the effects of radiation to the low-dose group (Moriyama and Kato 1973). This is very poor epidemiology since it assumes a linear or at least monotonic dose response for its validity.

The hormesis proponents’ overall argument is simplistic and straightforward.

1. The main target for radiation effects is the nuclear (or germ cell) DNA.
2. It is known that nuclear DNA is repairable and that the repair system efficiency is inducible by small doses of external radiation (up to 10 mSv).
3. It would seem likely that evolution would have developed such a protective effect, and this would be also predicted from natural selection considerations.
4. Therefore it should follow that small doses of external radiation will increase the surveillance and repair mechanisms (cell concentration of protective substances for reactive oxygen species, e.g., superoxide dismutase, etc.).
5. There is evidence that in animal studies a priming small dose of external radiation (gamma, X-ray) confers a protective effect on those individuals that are exposed to a second larger dose.
6. Nuclear worker and other studies based on external dose assessments show a reduction in effect when the lowest dose range is compared with higher doses, which the hormesis advocates present as evidence for their thesis.
7. Increasing the dose increases the damage *as measured by the end point*.

The simple answer to each point follows:

1. It is accepted that DNA is the target.
2. Inducible repair as exposure increases is accepted. But the concept of “dose” is not applicable to internal radionuclides, neither those that bind to DNA (Uranium, Radium, Strontium, Barium) nor to those that do not. The minimum dose to the cell from an average alpha particle track is upwards of 400 mSv, which is enormously greater than the doses that are suggested to be involved in inducible repair. Therefore even if the hormesis argument is accepted it can only apply to external radiation.
3. It would seem reasonable that evolution would have provided a mechanism for protecting against radiation. It has done so for ultraviolet radiation, which is also genotoxic.
4. But it does not follow that such a process is without a penalty; otherwise it would seem that the system should have evolved to be operating at full strength all the time and not in proportion to the external radiation level. The most obvious downside is that repairing involves a greater cell replication rate, and this would have a harmful effect on cell line longevity. It should be noted that the induction of suntanning does not reduce the incidence of skin cancer in high sunlight areas; that in reality, these areas have the highest rates. The authors do not discuss this.
5. Animal study evidence is accepted, but the authors assume that the zero dose level exists before the priming dose. This fails to address the background or pre-priming dose level of repair efficiency. Also, few studies have examined the low-dose region.
6. The clearest example of this failure is, in fact, in the very evidence that the authors believe supports their thesis, that is, the nuclear workers studies. The lowest dose range in the nuclear worker studies is 0–5 mSv. For example, the Cardis et al. (2007) study results show for most separate cancers a high effect per Sievert in the low-dose region with peak doses dependent on the type of cancer. The trend in risk peaks at the lowest doses and thereafter falls. But the low-dose group has been already exposed to radiation at a low level. And the true excess risk from cancer is unknown because the nuclear workers have a significant healthy worker effect. Often, this effect results in a negative excess relative risk, obtained by snapping a regression line across the observed dose response.
7. Increasing dose may increase damage but it does not follow that it increases the end-point measure since at some stage the cell or the developing embryo will be killed causing a reduction in the end point.

The real nature of the dose response relation at low doses has been studied in a large number of systems by E.B. Burlakova, director of the Radiobiology Committee of the Russian Academy of Sciences. She has published many studies of her findings, which show that the true dose response is biphasic (Burlakova 2000). That is, it increases from zero dose and then falls and then increases again. The authors fail to cite this work. This dose response is seen in many studies including all the nuclear worker studies. It is seen in the dose response for colon cancer in the A-bomb survivors. The increased cancer rate at low dose is real, and indeed is a big increase on cancer effect in a hypothetical healthy worker-adjusted population (Cardis et al. 2007). Burlakova’s explanation is that the true dose response is supralinear or saturates. Then the induction of repair at some point superimposed on this causes it to fall in the region of adequacy of the repair effect. But at the point that the dose is so high the repair ability is overwhelmed, the response rises again.

The alternative hypothesis is that the biphasic response results from the existence of two phases of cells, sensitive repair replication phase and insensitive quiescent phase. These phases are known in cell culture experiments to have 100-fold differences in radiation sensitivity (Busby 2013).

### Philosophy

The philosophical arguments in the article are based on the falsifiability concepts embedded in the writings of Kuhn and Popper. But these philosophy of science writings are based on a linear idea of the historical advances in science that have been addressed by the philosopher Paul Feyerabend in his book *Against Method* (2010), which the authors might do well to read. Feyerabend argues that every set of “facts” or observations can be interpreted equivalently well in different ways; the main interpretation that may be taken forward and form the basis of the kind of Kuhn processes may in fact not be the correct one for various reasons. Therefore the authors’ essays in philosophy are as questionable as the biological arguments they advance.

### Conclusion

Analysis based on linear assumptions (the LNT dose response) is invalid, neither is it protective. In this, the application of biology to the interpretation of epidemiology, I am fully in agreement with the authors. However the authors have not taken their method far enough. There are good reasons to believe that the high risk at low dose is real and represents a peak in effect which is due either (a) to the onset and overwhelming of repair induction or (b) to the existence of cells in two phases of activity, insensitive quiescence and repair replication. The authors might usefully examine Busby (2013) and Schmitz-Feuerhake et al. (2016).

The article is interesting and welcome in that it makes clear that the current radiation risk model is flawed by being based on severely limiting reductionist assumptions about the dose–response relationship, which both biological considerations and implausible correlation approaches clearly invalidate. However, the assumptions of the authors are similarly questionable and are the result of fitting a prior paradigm, that of “hormesis,” to those pieces of observational data that fit their chosen interpretation. A deeper investigation of the issue both biologically and philosophically reveals that not only are the authors in error in their attempt to increase the regulatory limits, but that the limits should be altered in the opposite direction because the true dose response is biphasic, and the hormesis argument is a misinterpretation of the falling part of the initial peak at low dose.

Finally, there is clear evidence of the effects of low internal doses: the health effects of the Chernobyl accident exposures. Whilst there have been widely differing analyses of adult health outcomes, there has been a consistent reporting of significant genetic effects as shown in birth outcomes and congenital malformations in Europe following the widespread contamination by fission products and uranium fuel particles. A recent review has collected together this evidence and interpreted it as showing a biphasic dose response with the highest heritable effects at very low doses, and doubling doses less than 10 mSv (Schmitz-Feuerhake et al. 2016).

## Response by the Authors to the Comments from Drs. Schmitz-Feuerhake and Busby on “Epidemiology Without Biology”

In our response to the two sets of comments we address each critique separately and add a general comment at the end.

**Schmitz-Feuerhake:**

We thank Dr. Schmitz-Feuerhake for her comments.

In her opening sentence she accuses us of attacking a straw man—“…there is no LNT hypothesis in radiation biology”—then pronounces this nonexistent hypothesis “just a practical approximation for radiation protection,” and later restates her denial in a different guise—“…no scientist claims that ‘*all* acute exposures to ionizing radiation are harmful…*regardless of how low the dose*.’” She then asserts, as though it were a substitute for LNT, that “the hypothesis is that ‘stochastic’ effects exist.”

On her first point, the LNT hypothesis pervades much of radiation science, including radiation biology—a hypothesis that, among other things, entails that all radiation is harmful no matter how low the dose or dose rate. Though our primary focus was epidemiology, it is the LNT hypothesis in radiobiology from which is derived a ubiquitous principle in the practice of radiology, that the minimum necessary exposure must be used, known as ALARA or as low as *reasonably* achievable—and in radiation regulatory policies, that the public and radiation workers must be protected against even the lowest *practically* achievable annual exposures.

LNT is central for leading radiobiologists, though some might not use the three-letter acronym. For example, Brenner et al. (2001), in a paper since cited over 1200 times and with an increasing frequency over the years, say the following:

The linear extrapolation without a dose threshold that is used to extrapolate cancer risks to very low doses has been the subject of much debate; however, the main regulatory and advisory groups that have reported on this issue have all concluded that the most scientifically credible approach to risk extrapolation to this dose range is a *linear extrapolation from greater doses, which is the assumption implicitly adopted here*. (2001, p. 294; emphasis added)

One of Brenner’s collaborators was Eric J. Hall, who also coauthored the textbook *Radiobiology for the Radiologist*. Even more explicit is the BEIR VII report cited in our article:

The committee concludes that current scientific evidence is consistent with the hypothesis that there is a *linear, no*-*threshold* dose–response relationship between exposure to ionizing radiation and the development of cancer in humans. (BEIR VII 2006, p. 323; emphasis added)

As to her counterposing stochastic effects to LNT, stochasticity is the fundamental thesis of the allegedly nonexistent LNT hypothesis and turns out to be its one valid aspect. But she presents it rhetorically as though we had denied it. Far from denying stochasticity, we affirm that the *damage* from radiation is both stochastic and linear. What makes the LNT hypothesis false is its neglect of the *biological response* to that damage by the organism.

Schmitz-Feuerhake’s substantive defense of the no-threshold (NT) aspect of LNT—i.e., the denial of hormesis, or the net beneficial effect—has two parts: First, without attempting to refute anything we say about radiation-caused carcinogenesis in the studies we did examine, she invokes two studies that we did not mention in our article, that of Canadian nuclear workers by Hazelton et al. and the 60-year-old work by Stewart, who studied cancer in children exposed to X-rays in utero. Second, she correctly but gratuitously notes that our “article regards only cancer and leaves out the hereditary risk” of low-dose radiation, pointing to several confirmatory studies that we also do not mention (Moller/Mousseau, Weinberg, Dubrova, Lasjuk). Since we did not focus our attention on hereditary effects, we did not include studies that provide evidence either for or against inherited effects of low-dose/low-dose-rate radiation. We did, however, mention in passing that our criticisms of LNT apply both to cancer causation and to hereditary effects.

While it is not incumbent upon us to discuss any of those studies, suffice it to say that four years after the 2007 Hazelton study the Canadian Nuclear Safety Commission (CNSC) reanalyzed the database on which Hazelton et al. relied and advised that it be withdrawn from further use by researchers until the inaccurate dosimetry could be corrected (CNSC 2011). Schmitz-Feuerhake neglects to mention this invalidation of the Hazelton study. Nevertheless, as she even states, Hazelton et al. found evidence of “…a protraction effect (sometimes called an inverse-dose-rate effect, where risk increases with protraction of a given dose).” From this, Schmitz-Feuerhake draws the conclusion that “any adaptive response—which is certainly dependent on dose-rate—will fail in cases of very low dose-rate.” She apparently does not realize that, put the other way around, this means that, at very low dose rate, risk *decreases* with *less* protraction of a given dose, i.e., at higher dose rates—a reflection of the *success* of the adaptive response, as well as hormesis, rather than failure.

This response is similar to that found by Sponsler and Cameron (2005), a study we cite in our article and discuss further in our response to Busby’s comments below—namely that nuclear shipyard workers with higher dose-rate exposures experience lower cancer rates and lower all-cause mortality (greater longevity) than their non-nuclear coworkers. Likewise, the Cohen (1990, 1995, 2004, 2008, 2010) study of radon and lung cancer that we cite and defend in our article, in which he discovered that the higher the radon concentration (i.e., the higher the dose rate of internal radiation exposure), the lower the lung cancer rates. Of course this is always within certain limits of dose rate, since at a sufficiently high dose rate the opposite occurs, and the organism suffers illness and death.

We did not examine the Stewart work, and have no comment on it. But Schmitz-Feuerhake invokes both this and the Hazelton study to imply that we had cherry picked the literature. What, we wonder, makes our selection of literature cherry picking but not hers? Suffice it to say, as we explain in our article, there is a decisive difference between cherry picking and selectivity. Since there are thousands of studies in this field, selectivity is unavoidable. In contrast, cherry picking is a *biased* selectivity, where reference is made only to studies that support one’s predilection while neglecting those that oppose it, and where reliance is placed on such studies without analyzing and identifying the errors in the opposing studies. Thus her accusation against us of cherry picking is misaimed, as we largely reference *precisely* studies that oppose our position. We do so in order to show how they consist of circular reasoning and therefore reach false and, as it turns out, dangerous conclusions. In contrast, she has not pointed to any error in our analysis, but rather has simply mentioned additional studies as though that alone negates our conclusions.

Furthermore, we justify our particular selection of targeted studies and policy statements on two grounds: first, that they currently occupy a prominent position in the field based on numerous recent favorable citations by advisory organizations and by other authors, and second, that a deeper analysis of a few illustrative examples would provide a tool enabling readers to engage in a similar reexamination of all past, present, and future studies that purport to demonstrate a causal relationship between low-dose/low-dose-rate radiation and illness, injury, or death—as well, incidentally, as hereditary damage.

Regarding Schmitz-Feuerhake’s point that LNT is “just a practical approximation for radiation protection,” there is indeed a distinction to be made between use of LNT for protection, from a policy perspective, and as a scientifically defensible hypothesis. This distinction is known as risk management versus risk estimation. Even regulatory agencies, as well as the International Commission on Radiological Protection (ICRP), National Council on Radiation Protection and Measurements (NCRP), and other advisory agencies and organizations, draw attention to this distinction and explicitly call for LNT *not* to be used for risk estimation but only for risk management. These sources emphasize this distinction because risk management, the key driver for policy setting, involves subjective value judgments in addition to the use of the LNT model. These value judgments include practicality, public sentiment, and economic and political considerations. Risk management is an effort to reduce risk or, as Schmitz-Feuerhake says, to protect the public through education and regulatory means. But a protection policy is only as legitimate as its weakest link. And policy makers defend LNT on the fallacious grounds that it protects by “erring on the side of caution.” As we demonstrate in our article, this is a dangerous illusion. To properly manage possible risk at low radiation doses, a range of possible health outcomes must be acknowledged, including beneficial (i.e., negative risk) or zero health effects. But use of the LNT model excludes such acknowledgment. That is why we spend a good portion of our article demonstrating that the use of LNT, even confined to risk management let alone estimation, has had harmful effects on hundreds of thousands, if not millions, of people. One cannot have it both ways. Either LNT accurately describes responses to low-dose/low-dose-rate radiation, or it doesn’t, and if it doesn’t—as we demonstrate—then its use as a basis of risk management is bound to have harmful effects, and in fact does.

Schmitz-Feuerhake, contrary to the LNT hypothesis, grants the existence of organismal response to repair the radiation-produced damage, but she argues that there is still residual harm—that even at low doses and dose rates the repair is at best partial, that there is “misrepair or failure of the immunological or adaptive response…,” and that “[it] is hardly biological understanding to believe that every instance of primary radiation damage will be completely compensated or lead to precautionary reactions in each individual, whether healthy, ill, predisposed, or temporarily distorted individual.” We deal with the concept of misrepair or failure of adequate defensive response in our article, and show that this incompleteness or failure, even if true, would almost certainly not yield a linear response as its advocates claim. More importantly, we show that damage to the cellular apparatus from the reactive oxygen species (ROS) generated by our normal metabolic processes occurs five to six orders of magnitude more frequently than that due to low levels of radiation, and that the effect of low-dose/low-dose-rate radiation is to stimulate such repair to the point that even much of the *spontaneous* endogenous damage is repaired and sufficient failures of repair removed to confer a net beneficial result on the exposed organism. Neglect of this far more significant damage and the beneficial effect on it of low-dose/low-dose-rate radiation is common to the writings of all LNT advocates that we have encountered.

In summary, perhaps the most important observation we can make is that Schmitz-Feuerhake *points to no actual erroneous substantive statement in our analysis* and instead mainly appeals, as a substitute for refutation, to the work of other authors. We enlarge on this approach below in our final paragraph.

**Busby:**

We thank Dr. Busby for his comments.

Points of his agreement with us include (a) that evolution would likely have provided protective mechanisms against at least some types of radiation, (b) that these mechanisms would likely be stimulated by those types of radiation, (c) that the LNT hypothesis is invalid, (d) that the introduction of underlying philosophical issues into scientific discourse is of value, and (e) that our criticism of those epidemiological radiation studies that neglect or distort biological considerations and evidence is a valid one—one that targets major flaws in radiation science that provide seeming support to the LNT hypothesis.

He also says he agrees with us that the target of radiation damage is DNA. While our article focuses more on DNA than on other molecules and cellular structures, we do mention in passing a point with which Busby seems implicitly to disagree, namely that there are also cellular targets other than DNA that suffer damage and, while they may or may not be repaired, nevertheless incite higher level organismal protections such as apoptosis (cell suicide) or immune system removal in the event of potential harm to the organism.

But then, contradicting his agreement with us that “It would seem reasonable that evolution would have provided a mechanism for protecting against radiation,” Busby charges us with proposing “a truly dangerous thesis because adopting the measures that [our article] suggests will result in a serious increase in illness and genetic damage in members of the public and workers who are currently protected under a regime which itself fails to take into account evidence (including that presented by the advocates of hormesis).” As we point out in our article, the dangers, and indeed deaths, that are a direct result of policies and fears based on the LNT hypothesis, and *that are not due to low*-*dose radiation exposure*, number in the hundreds to thousands. Busby is expressing his confidence in a thesis that shares the essential aspect of LNT—that all radiation is harmful no matter how low the dose or dose rate, i.e., without a threshold—albeit he rejects the linear aspect of the LNT hypothesis and insists that the harm at low doses is described by a bimodal curve and is even worse than LNT would predict, as we explain below. That is, Busby rejects the “L” in LNT but accepts the “NT,” no-threshold, aspect. And he, like the organizational, institutional, and individual targets of our critique, does so with no valid evidence to support his serious accusation, as we discuss below.

Busby next asserts that internal radiation differs from external radiation in important ways that, he charges, we neglect in our article. In particular, he states, “…the concept of ‘dose’ is not applicable to internal radionuclides…,” and in the next sentence he asserts, “The minimum dose to the cell from an average alpha particle track [the main form of radiation from radon and certain other isotopes] is upwards of 400 mSv, which is enormously greater than the doses that are suggested to be involved in inducible repair.”

There are several errors here. First, the concept of dose applies to any energy absorbed by tissue from ionizing radiation, regardless of its source’s location inside or outside the body, and in either case is defined in terms of the amount of energy absorbed per unit mass of tissue, usually expressed as joules per kilogram (J/kg). The unit of absorbed dose is the gray (Gy: 1 Gy = 1 J/kg). The dose from internally deposited radionuclides has for decades been routinely calculated for radiopharmaceuticals used in nuclear medicine diagnosis and treatment and is required by the U.S. Food and Drug Administration (FDA) for labeling. Indeed, after asserting that the concept of dose does not apply to internal exposures, Busby’s next sentence discusses dose in precisely that context.

Second, Busby’s use of the unit mSv (millisievert), instead of the mGy, for alpha particle traversals of single cells is invalid. The phenomenon of energy deposition in small targets pertains to the field of microdosimetry, and the units of mSv are not defined for microdosimetry. It is true that conventional internal dosimetric methods do not apply to alpha-emitting radionuclides, but the concept of dose is nevertheless still applicable. The product of a microdosimetric calculation is a statistical distribution of doses to small targets (e.g., cell, nucleus, etc.). An average dose can be determined, and it has been found to be meaningful as a predictor of response. For the average alpha energies and average cell sizes, the mean energy density imparted by a single track that randomly traverses a cell is in the range of 200–600 mGy. Additionally, single alpha tracks, depending on how directly their path intersects a cell, may impart smaller quantities of energy to the cell or cell nucleus, or even zero energy from near misses. Thus, Busby’s assertion that the “minimum” dose to the cell is 400 mSv is incorrect both quantitatively and qualitatively. This dose is more correctly stated as 400 mGy, and it is not the “minimum” dose to the cell, which could be as low as zero.

Third, even if it were true that a cellular dose of 400 mGy from internal alpha particles is “enormously greater than the doses that are suggested to be involved in inducible repair,” as Busby asserts, he neglects the different mechanisms (listed in our article and operable at and beyond 400 mGy) that protect the organism when DNA repair fails—some at the cellular, some at the tissue, and some at the organismal level. Repair is only one of two general categories of mechanism that protect the organism, the other being *removal* of the damaged cell from the organism—by means of apoptosis (cell suicide), bystander effect (murder by a neighbor), cleanup by the immune system, or direct destruction by the radiation itself (Feinendegen et al. 2012). With such removal, the energy deposition in a single cell by an alpha particle, while harmful to the cell, would have no deleterious effect on the organism.

Turning to a different accusation, Busby charges that we have neglected the natural background radiation dose received by subjects of epidemiological studies, and that we have falsely treated it as though it were zero dose. On the contrary, one of our key criticisms of the paper by Leuraud et al. is precisely that it is they who have neglected natural background radiation, as well as medical imaging exposures and other radiation sources, treating them as though they were zero, thus underestimating the putatively offending cumulative doses.

Another charge by Busby is that the neglect of natural background dose was also a flaw in the Sponsler and Cameron (2005) nuclear shipyard worker study that we cite to support our contention that hormesis exists. He then cites, as a counterexample, a different study of nuclear workers by Cardis et al. covering 15 countries. Aside from the fact that the reasoning in the Cardis study contains some of the same flaws as the studies by Leuraud et al. and Richardson et al. that we analyze in our article in some depth, the Cardis study is among those rendered invalid by the CNSC's reanalysis of the Canadian data, mentioned above. In the Cardis study Canadian data were single-handedly responsible for conferring statistical significance on their conclusion that cancer risk was due to the workers’ radiation exposure, so the removal of those data negates their conclusion. Neither Busby nor Schmitz-Feuerhake seems aware that the study’s invalidation by the CNSC is even subsequently acknowledged by Cardis and her coauthors, Richardson and Leuraud and colleagues, in Richardson et al. (2015), though that paper is Busby’s first reference:

INWORKS did not include data from Canada, a cohort for which the excess relative rate per Gy estimate was considerably larger than that observed in most other countries in the parent study, and for which concerns have been raised regarding data quality and completeness. (Richardson et al. 2015)

Despite this acknowledgment, however, Cardis et al. have not withdrawn their study.

Most importantly, Sponsler and Cameron, while also not taking account of the natural background and medical sources of radiation, focused on the fact that the dose *rates* differed systematically between the nuclear and non-nuclear workers. Thus only two specific average values of dose *rate*, rather than a continuum of values of cumulative dose, were involved—those for the workers dealing with the nuclear reactors in the studied shipyards and those who worked in the same shipyards distant from the reactors. Their use of dose rate as the independent variable renders Busby’s criticism irrelevant. By eliding the difference between studies based on cumulative dose and those based on dose *rate*, Busby in effect conflates two qualitatively different entities, a point we return to below.

In a further attempt to undermine Sponsler and Cameron’s conclusions Busby asserts that nuclear worker studies in general fail to take into account the healthy worker effect, which holds that workers capable of hard physical labor are healthier than average, and their lower illness rates, including cancer, are due to their better health rather than to exposure to chronic low-dose-rate radiation. However, this flaw was explicitly avoided by Sponsler and Cameron, as they deliberately used as a control group non-nuclear *shipyard* workers who were just as healthy on average as their nuclear coworkers.

Next Busby cites approvingly the work from the late 1990s and early 2000s by Russian researcher Elena Burlakova and her colleagues, asserting that their studies demonstrate “[t]he real nature of the dose response relation at low doses.” This work was based on both laboratory experiments and epidemiology of Chernobyl’s clean-up workers (“liquidators”). Burlakova et al. concluded that at least some, but not all, of the several observed dose–response relationships, but only at the cellular level, have a bimodal character that rises sharply from the baseline at zero dose to a high level, then falls almost as rapidly below the line postulated by the LNT hypothesis, and then rises again to essentially parallel the LNT-like relationship. It is this single observation from their *experimental* microscopic laboratory findings that Busby endorses and claims is also representative of the *epidemiological* relationship between cancer and cumulative dose, though Burlakova et al. found no such relationship at the organismal level, nor did all of their cellular-level relationships exhibit this pattern. In other words, Busby claims that radiation-caused carcinogenesis is even greater at low doses than the LNT hypothesis predicts. He also misses or neglects the fact that the dose *rates* that Burlakova and her colleagues studied in their laboratory experiments were three to four orders of magnitude (thousands of times) greater than natural background and other everyday sources of radiation, including those around Chernobyl, except perhaps in the first months after the accident when the radioactivity had not yet decayed away to any great extent.

Burlakova and her colleagues were ambivalent about drawing conclusions with respect to carcinogenesis and multiple other illnesses among the Chernobyl liquidators from their examination of microscopic changes in cell membrane lipids and other molecular cellular constituents. Yet Busby has selectively adapted as an *epidemiological* relationship their bimodal microscopic relationship, ignoring their other observed experimental dose–response relationships for different cellular features—those, for example, that exhibited non-bimodal or even inverted character. He also claims that it describes carcinogenesis regardless of whether the dose rate is low or very high. Moreover, since Burlakova’s relationships between cellular changes and cumulative dose were observed at fixed dose rates, cumulative dose becomes a surrogate for time—time since onset or termination of exposure in the laboratory. *So the variations are simply a measure of the time course of damage and repair*, and not a dose–response relationship. Busby, however, treats it as though the independent variable were a measure of acute dose—another conflation of cumulative dose and dose rate, and in this case a further conflation of dose and time. Furthermore, rather than a fixed dose rate, the liquidators, because they arrived at different times and because dose rates declined as time went on, experienced a wide variety of dose rates.

It should be noted that Busby was one of twelve members of an official UK committee called the Committee Examining Radiation Risks of Internal Emitters that, in an official report (CERRIE 2004), rejected by a vote of ten to two (Busby being one of the two dissenters) the applicability of Burlakova’s work to carcinogenesis. They did so on the following grounds:

that the data presented in the tables in Dr Burlakova’s studies were inconclusive as they could be read to indicate linear, biphasic or other responses. The data and their presentation also suffered from substantial shortcomings. For example, the selection of a single average to represent doses in epidemiological cohorts ignored the wide span of doses in each study. In addition, if the underlying response were biphasic, it would not have shown up in the studies, as the response would have been washed out by different individuals in each study having doses spread across the dose scale. (2004, p. 52)

Reference to the “wide span of doses” echoes one of our criticisms of the Leuraud study in our article.

Whether the majority were right or wrong, it is disingenuous for Busby to cite Burlakova’s work as a hurdle over which we need to jump. In the face of his accusation that we cherry picked our sources (a point we discuss further below), this citation, as well as his choice of only one of several observed dose–response relationships (relevant or not), is ironic, if not self-negating.

In his conclusion, Busby recommends that we “might usefully examine references Busby (2013) and Schmitz-Feuerhake et al. (2016),” the former authored by him alone and the latter by him along with our other commenter Schmitz-Feuerhake and a third author. Both include the same schematic bimodal curve, and the latter includes a scatter plot of data (Fig. 3) purportedly representing infant leukemia rates after the Chernobyl accident as a function of radiation dose (not dose *rate*) in four carefully selected countries—UK, Germany, Greece, and Belarus. The plot consists of nine points—all without confidence intervals, the same erroneous practice criticized by CERRIE in the quote above as “the selection of a single average to represent…the wide span of doses”—two each for UK, Germany, and Belarus (“high” and “low” *dose*) and three for Greece (“high,” “intermediate,” and “low” *dose*). But, unlike the other three, Germany’s greater effect is in the “low” (dose) area, an inconsistency that the authors overlook. Though we can expect beforehand that almost certainly there are overlooked variables that differ from place to place, Germany’s reversal alone serves as evidence for confounding by these variables. So this plot consists of points that have no meaning for three reasons—a false independent variable (dose rather than dose rate), unwarranted precision (no confidence intervals), and overlooked confounding. *But the points appear to lie along a bimodal curve*, indicating that these meaningless points were cherry picked for just that reason. Additionally, in general the geographic regions on earth with the highest dose *rates* experience no greater, and often lower, cancer rates than those with the lowest dose rates (Dobrzyński et al. 2015; see our article).

Finally, Busby applauds our reference to philosophy. However, he disapprovingly opines that our philosophy of science derives from Popper (whom we never mention in our article) and Kuhn (whom we mention once in passing). He then incorrectly lumps them together as falsificationists, though only Popper is a falsificationist. He also asserts that both of them argue for the linear progress of scientific truth, though Kuhn at times repudiates progress and at other times is ambiguous on that issue. But this is a discussion for another time and place, one that we mention only to indicate Busby’s philosophical errors in addition to his factual ones concerning radiation.

Interestingly, Busby instead refers us to the work of Feyerabend, thereby choosing a philosophy that removes all foundation from his factual claims, because, in Busby’s paraphrasing, Feyerabend argues “that every set of ‘facts’ or observations can be interpreted equivalently well in different ways.” If Feyerabend were correct in this, there would be no basis for Busby to disagree with us, let alone pronounce us wrong.

Furthermore, as a corollary, Busby’s accusation that we are guilty of cherry picking, if it means anything, is a charge that we neglect to mention studies whose conclusions oppose ours and confine ourselves to citing only studies that support our contentions. However, this accusation presupposes an affirmation by Busby that there are indeed studies that *cannot* “be interpreted equivalently well in different ways,” but rather are *more*, or *less*, valid. Thus Busby’s claims both about radiation science and philosophy are either invalid or inconsistent, or both.

Busby concludes, incoherently given his support of Feyerabend, with the comment that “the assumptions of the authors are similarly questionable and are the result of fitting a prior paradigm, that of ‘hormesis’ to those pieces of observational data that fit their chosen interpretation.” Since our central contention is that there exists radiation dose and dose-rate thresholds below which there is no harm to the organism (and indeed there is benefit—hormesis), the “pieces of observational [and experimental] data that fit [our] chosen interpretation” are precisely those that reveal the very hormetic range that the authors of so many epidemiological studies exclude and, based on the exclusion, deny exist. The paradigm that includes hormesis is not a “prior paradigm,” but rather, in Bayesian terminology, is a “posterior” or an a posteriori paradigm, derived from a *more* inclusive assessment of nature (i.e., selective but not cherry picked) rather than a less inclusive one (i.e., cherry picked). The less inclusive paradigm and its longstanding *politically* dominating status mislead many investigators into obscuring the existence of hormesis in the low-dose, low-dose-rate ranges and arriving, unwittingly or not, through circular reasoning at the LNT hypothesis. The hormetic aspect only becomes visible by examination of the broader epidemiological domain and, most particularly, in the biological domain.

**Addressed to both sets of comments:**

Readers of scientific studies, in this field among many, have to remain continually vigilant against the all-too-common strategy employed by both Schmitz-Feuerhake and Busby: rather than rely on refutations of specific points, they change the subject, invoking study after study, implying that these suffice to demonstrate our errors. We have tried to show how a class of epidemiological studies in radiation science—those that deny hormesis and the existence of dose and dose-rate thresholds—are rooted in the failure to appeal to biology for either the source of their hypotheses or validation of their conclusions, or both. We analyze mainly studies that deal with external radiation and with carcinogenesis rather than with hereditary aspects. Neither of our critics has pointed to any actual flaws in our reasoning and have both brought up aspects that are not part of our analysis. This is not a scientific approach, in which the goal should be to arrive at a greater comprehension of reality rather than a further confirmation of a favored paradigm.

## Footnotes

1 Editor’s note: Because this article drew strong responses, both positive and negative, we thought exploration of its important topic would be best served by open debate. To this end, the authors agreed when the paper was submitted to the review process to having opposing views published alongside their own. We sought out anonymous peer reviewers for the article as usual but offered the possibility of then publishing their critiques; two reviewers kindly agreed to relinquish their anonymity to do so. Their commentaries are included at the end of this article, followed by a rejoinder by the authors.

2 The conversion from Gy to Sv involves several factors, including the impact of different types of radiation and the nature of the tissue receiving the radiation. The conversion also involves some of the very assumptions about risk that we dispute in this article. It is generally agreed that for gamma rays and x-rays and for whole body exposure the numerical conversion factor is unity. Thus, for those types of exposure we use either Gy or Sv depending on the usual unit used in the particular context or the unit used by the authors of quoted material.

3 A link to the audio recording is available on the article’s website: http://thelancet.com/journals/lanhae/article/PIIS2352-3026(15)00094-0/fulltext (accessed 15 April 2016).

4 Department of Physics, University of Bremen, Bremen, Germany; e-mail: ingesf@uni-bremen.de.

5 Environmental Research SIA, Latvian Academy of Sciences, Riga, Latvia; e-mail: christo@greenaudit.org.
